# Unravelling Hypoxia Tolerance: Transcriptomic and Metabolic Insights From *Lucinoma capensis* in an Oxygen Minimum Zone

**DOI:** 10.1111/mec.70194

**Published:** 2025-12-01

**Authors:** Inna M. Sokolova, Eugene P. Sokolov, Helen Piontkivska, Stefan Timm, Katherine Amorim, Michael L. Zettler

**Affiliations:** ^1^ Department of Marine Biology, Institute for Biological Sciences University of Rostock Rostock Germany; ^2^ Department of Maritime Systems, Interdisciplinary Faculty University of Rostock Rostock Germany; ^3^ Department of Biological Sciences Kent State University Kent Ohio USA; ^4^ Brain Health Research Institute Kent State University Kent Ohio USA; ^5^ Department of Plant Physiology, Institute for Biological Sciences University of Rostock Rostock Germany; ^6^ Department of Biological Oceanography Leibniz Institute for Baltic Sea Research Warnemünde Rostock Germany

**Keywords:** adaptation, metabolism, metabolomics, oxygen fluctuations, transcriptomics

## Abstract

The lucinid clam *Lucinoma capensis* thrives at the oxygen minimum zone margins in the Benguela Upwelling System, where oxygen levels fluctuate dramatically. Understanding its adaptation to such extreme conditions provides key insights into survival strategies under fluctuating oxygen availability. We investigated the transcriptomic and metabolomic responses of *L. capensis* under normoxia, hypoxia, and recovery, focusing on the gills and digestive gland. Our findings highlight distinct organ‐specific responses, with the gills showing strong transcriptional changes to oxygen fluctuations, in contrast to the more stable profile observed in the digestive gland. Under hypoxic conditions, the gills exhibited coordinated downregulation of protein synthesis, transposable element activity, and immune function, suggesting a tightly regulated energy conservation strategy and mechanisms to preserve symbiont stability and genomic integrity. Activation of prokaryotic metabolism in the gills supports the symbionts' role in host energy acquisition and sulfide detoxification during hypoxia. In contrast, the digestive gland showed minimal transcriptional shifts during anoxia, with upregulation of pathways supporting structural maintenance. Upon reoxygenation, the gills displayed an active and asymmetric recovery, characterised by rapid restoration of protein synthesis and gradual normalisation of protein degradation and immune functions. Despite significant transcriptomic changes, the metabolome remained largely stable, reflecting *L. capensis* 's resilience to oxygen fluctuations. However, an overshoot in TCA cycle intermediates and derepression of previously downregulated pathways indicate that reoxygenation involves active metabolic reprogramming, not merely a return to baseline. This study highlights the specialised tissue responses and symbiotic contributions that enable *L. capensis* to thrive in one of the ocean's most challenging environments.

## 1 Introduction

Since the Great Oxidation Event over 2.2 billion years ago, oxygen (O_2_) has shaped the evolution of complex life and biodiversity (Lane 2002). It remains central to the survival of most multicellular organisms through its role in cellular bioenergetics (Lane 2005; Schmidt‐Rohr 2020). Fluctuations in oxygen availability, such as hypoxia and reoxygenation, impose major stress: hypoxia disrupts oxidative phosphorylation, reducing ATP production and shifting metabolism towards less efficient anaerobic pathways (Hochachka and Mustafa 1972; Schreurs et al. 2007), while reoxygenation often generates reactive oxygen species (ROS), causing oxidative damage (Halliwell and Gutteridge 1999; Hermes‐Lima 2004). These fluctuations impact not only energy metabolism but also broader transcriptional and metabolic networks, requiring diverse adaptive responses across species and habitats (Chen et al. 2024; Connor and Gracey 2012; Gracey et al. 2008; Lockwood et al. 2015; Yang et al. 2023). Coastal and upwelling regions are especially prone to hypoxia, driven by eutrophication, organic matter decomposition, and restricted oxygen replenishment (Howarth et al. 2011; Scholz 2018). Climate change exacerbates these trends by reducing oxygen solubility and fuelling respiration, which accelerates deoxygenation and biodiversity loss (Breitburg et al. 2019; Deutsch et al. 2024). Oxygen minimum zones (OMZs) exemplify these dynamics, where nutrient‐rich upwelling enhances productivity but depletes subsurface oxygen (Scholz 2018).

The Benguela Upwelling System (BUS) is a prominent OMZ with extreme variability, including seasonal hypoxia, hydrogen sulfide accumulation, and high organic matter degradation (Amorim and Zettler 2023; Mohrholz et al. 2008). Oxygen levels range from well‐oxygenated in late winter and spring to hypoxic or anoxic in late summer and autumn, with pronounced fluctuations along the OMZ margins (Zettler et al. 2013). Despite these stresses, the BUS margins support a productive ecosystem dominated by molluscs and polychaetes. The bivalves *Lucinoma capensis* and *Lembulus bicuspidatus* are particularly abundant, thriving in sulfide‐rich muds under harsh and fluctuating conditions (Amorim et al. 2021; Eisenbarth and Zettler 2016; Zettler et al. 2013). Although the exact age of the BUS OMZ is uncertain, evidence suggests the upwelling system has persisted for over 3 million years (Mohanty et al. 2024), providing ample time for the evolution of unique physiological and molecular adaptations. Understanding these mechanisms is essential for explaining resilience in OMZ ecosystems and for anticipating the broader impacts of ocean deoxygenation on marine biodiversity.

The lucinid clam *L. capensis* exhibits a remarkable capacity to thrive in the hydrogen sulfide‐rich sediments of the BUS OMZ, with populations reaching biomass levels of up to 300 g m^−2^ (Amorim et al. 2022). Similar to other lucinids, *L. capensis* forms a symbiotic association with sulfur‐oxidising gammaproteobacteria (*Candidatus Thiodiazotropha*), which detoxify sulfide and supply essential nutrients, enabling the host to inhabit this extreme environment (Amorim et al. 2022; Cary and Felbeck 1989; Osvatic et al. 2023). While the ecological and symbiotic adaptations of lucinid clams have been extensively studied (Amorim et al. 2022; Cary and Felbeck 1989; Lim et al. 2019; Osvatic et al. 2023; Yuen et al. 2019), the molecular and physiological mechanisms underlying their responses to severe oxygen fluctuations remain poorly understood.

This study addresses this critical knowledge gap by investigating the transcriptomic and metabolomic responses of *L. capensis* to normoxia (~6.9 mL O_2_ L^−1^), severe hypoxia (~0.11 mL O_2_ L^−1^ for 36 h), and post‐hypoxic recovery (1–24 h of normoxia following hypoxia). Two key host tissues were analysed: the gill, which facilitates gas exchange and hosts sulfur‐oxidising bacterial symbionts, and the digestive gland, which is central to energy storage and metabolism (Amorim et al. 2022; Lim et al. 2019; Yuen et al. 2019). Unlike most previous studies, which primarily focus on the ecological roles and symbiosis of OMZ organisms (Amorim et al. 2022; Cary and Felbeck 1989; Lim et al. 2019; Osvatic et al. 2023; Yuen et al. 2019), this research provides a rare, in‐depth investigation of the host's molecular and metabolic adaptations to fluctuating oxygen conditions. By examining transcriptomic and metabolomic changes across these conditions, this study provides novel insights into the adaptive strategies that enable *L. capensis* to survive and thrive in the dynamic and extreme environment of the BUS OMZ. The focus on reoxygenation stress—a condition often overlooked but critical for organisms in such habitats—further enhances our understanding of the molecular and physiological mechanisms that mediate the environmental resilience of *L. capensis* in OMZ ecosystems.

## 2 Materials and Methods

### 2.1 Chemicals

All chemicals and enzymes were obtained from Biozym Scientific GmbH (Hessisch Oldendorf, Germany), VWR (Darmstadt, Germany), or Fisher Scientific (Schwerte, Germany), and were of analytical grade or higher.

### 2.2 Animal Collection and Maintenance

**FIGURE 1:**
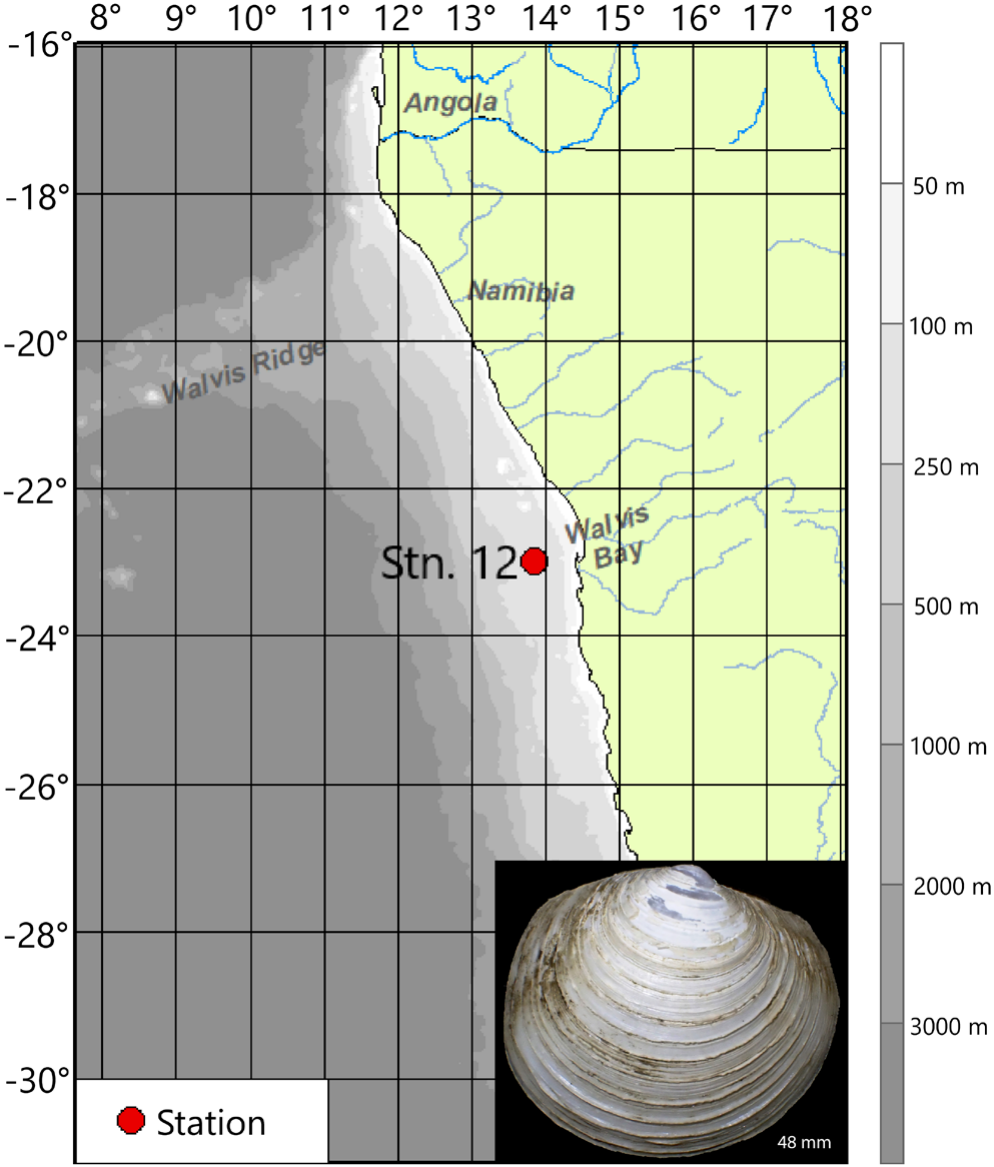
Map of the collection site and *Lucinoma capensis* photo (insert).

Adult *L. capensis* (2.1 ± 0.7 cm) was collected at a depth of 150 m by dredging in the central region of the Namibian shelf onboard the FS Meteor during the EVAR Expedition M157 (Figure 1). A sampling and research data use permit was granted by the National Commission on Research, Science and Technology, Namibia (permit RPIV00812019). *L. capensis* is not a species covered by the Convention on Biological Diversity and the Convention on International Trade in Endangered Species of Wild Fauna and Flora. During collection, temperature, salinity, and oxygen concentration in the benthic boundary layer were measured using the CTD‐system “SBE 911plus” (Seabird‐Electronics, USA). In the BUS, *L. capensis* is commonly found in areas of highly variable oxygenation, with dissolved oxygen concentration varying from values < 0.2 to < 3.4 mL L^−1^ (Amorim and Zettler 2023). Our collection took place during the austral spring (August–September), with oxygen concentrations in *L. capensis* habitats ranging from 0.73 mL O_2_ L^−1^ to 2.4 mL O_2_ L^−1^ during sampling (Amorim and Zettler 2023). Animals used in this study were collected at approximately 11.7°C, and a dissolved oxygen concentration of 0.73 mL O_2_ L^−1^. All animals used in the experiment were collected in a single dredging from station 12 (23.00° S, 13.87° E, Figure 1).

### 2.3 Experimental Exposures

Following dredging, 59 individuals of *L. capensis* were randomly distributed into twelve 500 mL glass containers with in situ water at 10°C (1.3 μmol PO_4_ L^−1^; 24.9 μmol NO_3_ L^−1^; 0.26 μmol NO_2_ L^−1^; 0.73 μmol NH_4_ L^−1^; 31.1 μmol SiO_2_). Nutrient concentrations in the water were measured in an auto‐analyser (QuaAtro, Seal analytical) using standard calorimetric methods (Grasshoff et al. 2009). Because the bivalves sampled were of highly variable sizes, each bottle received 4–6 individuals, aiming that the sum of the shell lengths of the individuals in each bottle was ca. 10 cm. Twenty hours after sampling, the bottles with the bivalves were assigned to four different conditions (three replicate containers per condition): normoxia (36 h at 6.9 ± 0.24 mL O_2_ L^−1^), severe hypoxia (36 h at 0.11 ± 0.05 mL O_2_ L^−1^), 1 h or 24 h of reoxygenation (36 h of severe hypoxia followed by 1 or 24 h of normoxic recovery). Water in the normoxic treatments was aerated with ambient air. Hypoxia was achieved by continuously bubbling nitrogen through the buffered seawater. O_2_ concentrations were measured using an Optical Oxygen Meter (FireStingO2, Aachen, Germany). All exposures were performed in a temperature‐controlled room at 10°C in the dark. Nutrient concentrations were measured in water by the end of exposures in one bottle of hypoxia condition (2.7 μmol PO_4_ L^−1^; 20.2 μmol NO_3_ L^−1^; 2.2 μmol NO_2_ L^−1^; < 0.5 μmol NH_4_ L^−1^; 34.5 μmol SiO_2_) and two bottles of normoxic condition (0.5–1.4 μmol PO_4_ L^−1^; 5.9–19.3 μmol NO_3_ L^−1^; 2.6–3.8 μmol NO_2_ L^−1^; < 0.5 μmol NH_4_ L^−1^; 32.3–33.5 μmol SiO_2_). Except for the lower ammonium levels, other nutrient concentrations were similar to the initial levels in the habitat water.

All bivalves were alive after exposure and were snap frozen and stored at −80°C. Frozen tissues (−80°C) were delivered to the Leibniz Institute of the Baltic Sea Research in Rostock and then transported on dry ice to the Novogene Sample Receiving Department (Cambridge, U.K.).

### 2.4 Transcriptome Sequencing and Gene Expression Analysis

The RNA extraction, polyA enrichment, mRNA library preparation and next‐generation (Illumina NovaSeq 6000 PE150) sequencing were carried out by Novogene GmbH (Munich, Germany). Details of the sequence assembly are provided in Text S1. For the gill tissue, the sample size (*N*) was 6 for all experimental conditions. In the digestive gland, sample sizes were *N* = 2 for the normoxic condition and *N* = 3 for both the severe hypoxia and 24‐h recovery groups. The difference in sample size between the two tissues was due to both biological and technical constraints, including limited tissue availability and RNA quality issues that led to the exclusion of some samples. Budget constraints also limited sequencing, so we prioritised gill tissue, known to be most sensitive to hypoxia in bivalves (Lumor et al. 2025; Montúfar‐Romero et al. 2024), which was the primary focus of our transcriptomic analysis.

Gene expression levels were estimated with RSEM (Li and Dewey 2011), converted into FPKM (Fragments Per Kilobase of transcript sequence per Million base pairs sequenced), and annotated using BLAST (Altschul et al. 1997) as described in Text S1. Differential gene expression analysis was performed with DESeq2 (Love et al. 2014), using read counts as input data. Benjamini–Hochberg correction (Benjamini and Hochberg 1995) was used to adjust for multiple tests, with the cut‐off p‐adjusted value of 0.05 and |log2FoldChange| > 0.58 to identify differentially expressed genes (DEGs) that showed at least 50% change in expression level in either direction. Gene functional enrichment analysis was performed as described in Text S1 using three complementary approaches: Gene Ontology (GO) enrichment analyses as implemented in GOseq (https://bioconductor.org/packages/release/bioc/html/goseq.html) (Young et al. 2010), Reactome analysis (Milacic et al. 2023), and manual curation of annotated DEG database (Table S1).

It is important to note that the library preparation for RNA sequencing was performed using a poly‐A enrichment protocol, which selectively captures eukaryotic transcripts and can result in underrepresentation of bacterial mRNAs. Nevertheless, due to the high abundance of symbiotic and associated bacteria in gill tissues, a substantial number of prokaryotic transcripts were detected (Table S3). In contrast, the digestive gland—lacking intracellular symbionts and containing only a typical extracellular microbiome—showed no detectable prokaryotic differential expression. However, without targeted microbial metatranscriptomics or complementary metagenomic sequencing, we cannot confidently assign these transcripts to specific microbial taxa. As such, our interpretation of prokaryotic gene expression is limited to broad functional trends rather than taxon‐specific insights.

### 2.5 Metabolite Analysis

Targeted metabolome analysis was conducted using high‐performance liquid chromatography‐mass spectrometry as described in Text S2. In total, 35 metabolites—including amino acids, intermediates of the tricarboxylic acid (TCA) cycle and urea cycle, adenosine monophosphate (AMP), S‐adenosylmethionine, carnitine, nicotinamide adenine dinucleotide (NAD) and γ‐aminobutyric acid (GABA)—were measured. Concentrations of iso(citrate), aconitate and fumarate were below the detection limits of the method. For the metabolomic analysis, sample sizes (N) in gill tissue were as follows: 8 for normoxia, 11 for severe hypoxia, 7 for 1‐h recovery, and 5 for 24‐h recovery. In the digestive gland, sample sizes were *N* = 8 for normoxia, 11 for severe hypoxia, 8 for 1‐h recovery, and 7 for 24‐h recovery.

Data distribution and variance were assessed using normal probability plots and Bartlett's test. For succinate levels in gills, zero values were replaced by ½ of the lowest positive value considered a limit of detection (4.21 ng mg^−1^). Data for all metabolites except AMP met the criteria for normality and homogeneity of variances and were analysed via one‐way ANOVA (oxygen condition as a fixed factor), followed by Tukey's Honest Significant Differences test for post hoc comparisons. For AMP concentrations, the non‐parametric Kruskal‐Wallis test was applied, with Dunn's test used for multiple comparisons. The ANOVA and Kruskal‐Wallis analyses were conducted using GraphPad Prism version. 7.05 (GraphPad Software Inc., La Jolla, CA, USA).

Multivariate analyses of the metabolite profiles, including sparse partial least squares—discriminant analysis (sPLS‐DA), correlation analysis and clustering, were performed on data that were autoscaled (mean‐centred and divided by the standard deviation of each variable) to minimise the effects of differences in absolute metabolite concentrations as implemented in MetaboAnalyst 6.0 (Pang et al. 2024). The sPLS‐DA algorithm is well‐suited for datasets where the number of variables (metabolites) exceeds the number of samples (Lê Cao et al. 2011). In our model, we specified a maximum of five principal components, with 10 variables selected per component. The effects of oxygen condition on tissue‐specific metabolism were assessed using pathway enrichment analysis in MetaboAnalyst 6.0 (Pang et al. 2024). The *Drosophila melanogaster* metabolic reference library and the Globaltest method were used for pathway enrichment, with relative betweenness centrality as the measure of node importance. Pathways with a false discovery rate (FDR) < 0.05 and a pathway impact > 0 were considered for further analysis. The metabolomics data are available via Zenodo, https://doi.org/10.5281/zenodo.15268760.

## 3 Results

### 3.1 Transcriptome Overview

**FIGURE 2:**
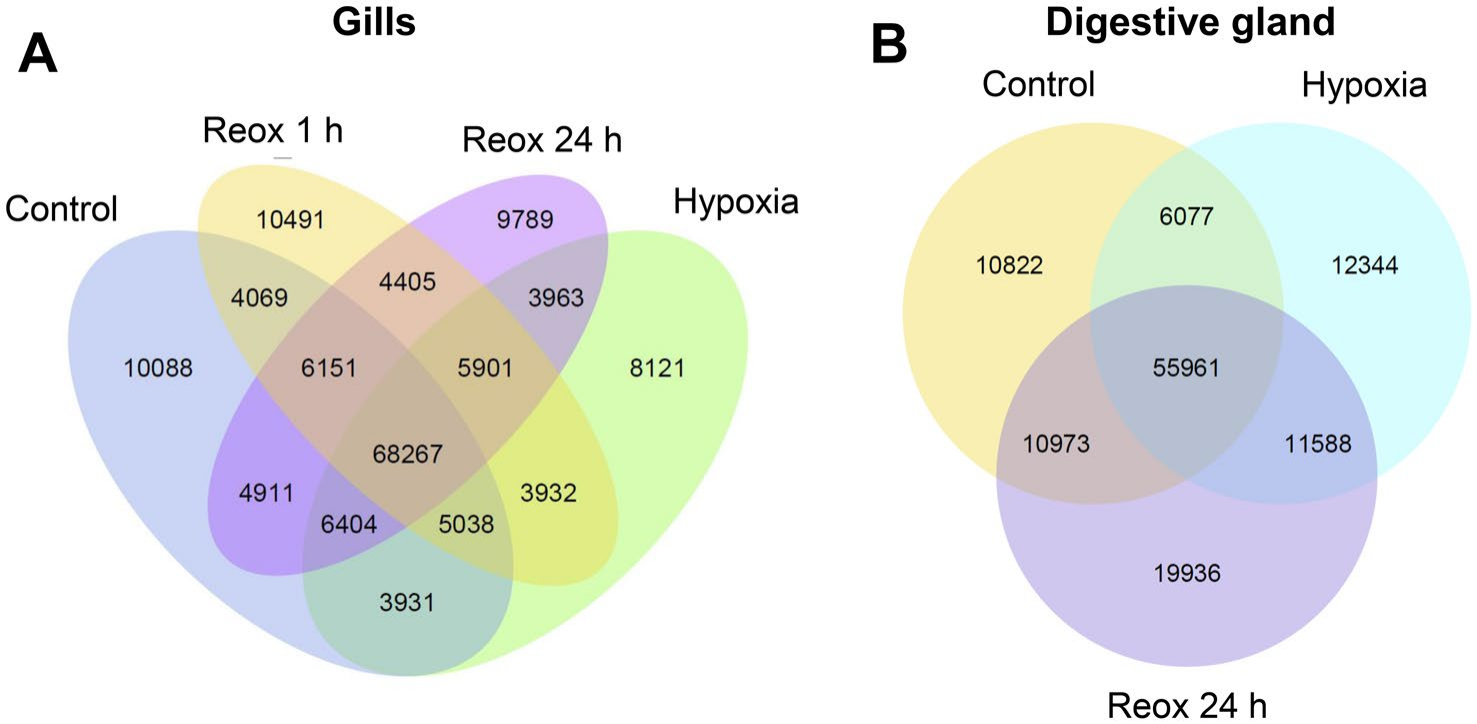
Venn diagram of the gill (A) and the digestive gland (B) transcriptome of *L. capensis* . Reox—reoxygenation. Numbers inside the respective sections indicate the number of uniquely expressed or co‐expressed transcripts.

A comparison of the whole transcriptome data across all replicates revealed a substantial number of co‐expressed transcripts among different exposure conditions in both studied tissues. Specifically, 68,267 transcripts in the gills and 55,981 transcripts in the digestive gland were shared across all treatments (Figure 2). In the gills, the highest number of uniquely expressed transcripts was observed after 1 h of reoxygenation (10,491), followed by the normoxic controls (10,088), 24 h of reoxygenation (9789), and the lowest during hypoxia (8121). In the digestive gland, the greatest number of uniquely expressed transcripts occurred in the 24‐h recovery group (19,936), followed by the hypoxia group (12,344) and the normoxic control (10,822).

### 3.2 Differential Gene Expression: Summary

**FIGURE 3:**
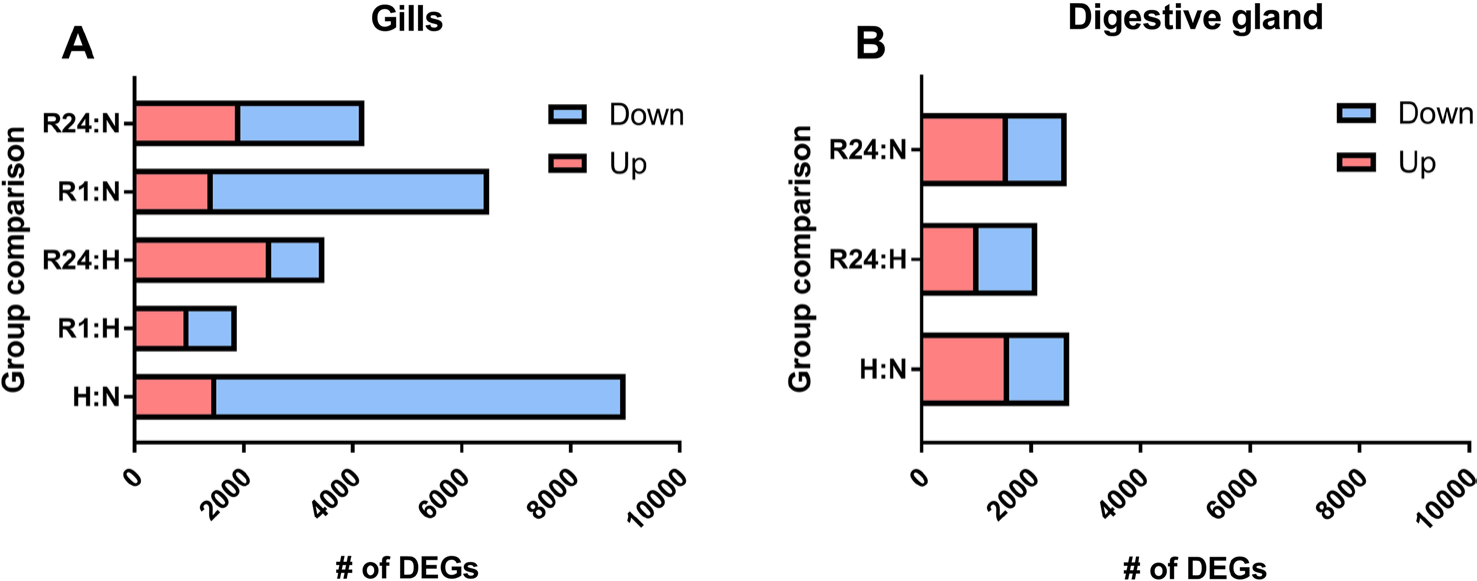
DEG counts in the gills (A) and the digestive gland (B) of *L. capensis* . Experimental conditions: H—hypoxia, N—normoxic control, R1—1 h of post‐hypoxia recovery, R24—24 h of post‐hypoxia recovery. The DEGs are shown for all pairwise comparisons of oxygen regimes within the two studied tissues.

Using the selected cut‐off criteria (*p*_adj_ < 0.05, |log2FoldChange| > 0.58), the highest number of DEGs, totaling 9006, was found when comparing the gill transcriptome of *L. capensis* under normoxic conditions to that of the clams exposed to hypoxia. Of these DEGs, 1500 were upregulated, and 7506 were downregulated (Figure 3A). After 1 h of post‐hypoxia recovery, the gill transcriptome remained significantly altered compared to the normoxic state, with 1436 upregulated and 5065 downregulated transcripts. Following 24 h of reoxygenation, 4214 gill transcripts were differentially expressed relative to the normoxic controls, with 1942 upregulated and 2272 downregulated.

In the digestive gland, 2697 genes (1597 upregulated and 1100 downregulated) were differentially expressed after hypoxia, and 2653 genes (1578 upregulated and 1075 downregulated) were observed after 24 h of recovery compared to the normoxic controls (Figure 3B).

Many of the identified DEGs were annotated as genes with unknown functions and were therefore excluded from further analysis.

### 3.3 Differential Gene Expression: Gills

In the hypoxic gills of *L. capensis* , 819 eukaryotic host transcripts were downregulated, while 236 were upregulated compared to normoxic controls (Table S2A,B). Among the downregulated transcripts, processes contributing more than 5% to the DEG pool included protein synthesis and breakdown, mobile genetic elements, immunity, and signalling. Conversely, the upregulated transcripts with greater than 5% representation were associated with proteolysis, transcription regulation, immunity, cytoskeleton organisation, carbohydrate metabolism, DNA maintenance, extracellular matrix, and protein synthesis. A marked imbalance was observed among functional groups, with protein synthesis showing a 12.5‐fold excess of downregulated over upregulated DEGs, followed by mobile genetic elements (8.3‐fold), signalling (5.9‐fold), and immunity (3.1‐fold).

During the recovery phase after 1 h of post‐hypoxia reoxygenation, 409 eukaryotic host transcripts were downregulated, and 289 were upregulated (Table S2C,D). The primary functional categories (> 5% of DEGs) among the downregulated transcripts were proteolysis, mobile genetic elements, immunity, transcription regulation and protein synthesis. The upregulated transcripts were mostly associated with proteolysis, extracellular matrix, mobile genetic elements, immunity, transcription regulation, and signalling. Apoptosis exhibited the most pronounced downregulation, with an over 8‐fold excess of downregulated compared to upregulated transcripts, whereas the extracellular matrix showed a 3.2‐fold excess of upregulated transcripts.

Following 24 h of reoxygenation, 261 eukaryotic host transcripts were downregulated, while 328 were upregulated (Table S2E,F). Among the downregulated DEGs, the most prominent (> 5%) functional groups included immunity, proteolysis, protein synthesis, and mobile genetic elements. The upregulated DEGs were predominantly associated with proteolysis, transcription regulation, DNA maintenance, mobile genetic elements, and signalling. The most substantial upregulation was observed in DNA maintenance, with a 3.8‐fold excess of upregulated over downregulated transcripts.

Due to the use of polyA enrichment during library preparation, which selectively captures eukaryotic transcripts, bacterial transcripts lacking polyadenylation may be substantially underrepresented in our dataset. As such, the prokaryotic DEGs reported here likely reflect only a partial view of the symbiont's transcriptional response. Nevertheless, for prokaryotic symbiont transcripts detected in the hypoxic gills, 40 were downregulated and 139 were upregulated compared to normoxic conditions (Table S3A,B). Downregulated transcripts (> 5% of DEG) were primarily associated with antibiotic and amino acid metabolism, and proteolysis. Among the upregulated prokaryotic DEGs, the most significant functional groups included sulfur metabolism, CO_2_ fixation, electron transport system (ETS), protein synthesis, and amino acid metabolism. Notably, the greatest upregulation compared to downregulation was observed in ETS (16‐fold), protein synthesis (15‐fold), and CO_2_ fixation (9‐fold).

After 1 h of reoxygenation in the gills of *L. capensis* , 88 prokaryotic symbiont transcripts were downregulated, while 52 were upregulated (Table S3C,D). The downregulated transcripts were primarily involved in amino acid metabolism, protein synthesis, and mobile genetic elements. In contrast, upregulated transcripts were predominantly associated with CO_2_ fixation, nitrogen and sulfur metabolism, transcription regulation, the tricarboxylic acid cycle, and amino acid metabolism.

Following 24 h of reoxygenation, 18 prokaryotic symbiont transcripts were downregulated, and 84 were upregulated (Table S3E,F). No dominant functional groups were identified among the downregulated DEGs, as most consisted of only 1–2 transcripts. The upregulated transcripts were enriched in protein synthesis, CO_2_ fixation, transcriptional regulation, sulfur metabolism, ETS, and chaperones, along with amino acid metabolism.

### 3.4 Differential Gene Expression: Digestive Gland

After hypoxia, we identified and functionally annotated 167 downregulated and 382 upregulated eukaryotic DEGs in the digestive gland of *L. capensis* compared to the normoxic control (Table S4A,B). Among the downregulated DEGs, the most prominent functional categories (> 5% of DEGs) were related to protein synthesis, mobile genetic elements and proteolysis. The upregulated DEGs were primarily associated with immunity, proteolysis and extracellular matrix. The largest excess of upregulated over downregulated transcripts was observed in the functional categories related to immunity (8.9‐fold) and proteolysis (3.5‐fold).

In the digestive gland of *L. capensis* following 24 h of reoxygenation, 144 transcripts were downregulated, while 384 were upregulated compared to normoxic conditions (Table S4C,D). The most prominent downregulated functional categories included protein synthesis, immunity, and mobile genetic elements. In contrast, upregulated transcripts were predominantly associated with protein synthesis, immunity, proteolysis, and neural function. Among these, the functional category related to proteolysis exhibited the most pronounced imbalance, with a 3.6‐fold excess of upregulated over downregulated transcripts. No prokaryotic DEGs were identified in the digestive gland of *L. capensis*, consistent with the use of polyA enrichment and the known lack of endosymbionts in this tissue (Amorim et al. 2022).

### 3.5 GO and KEGG Pathway Enrichment Analysis

**FIGURE 4:**
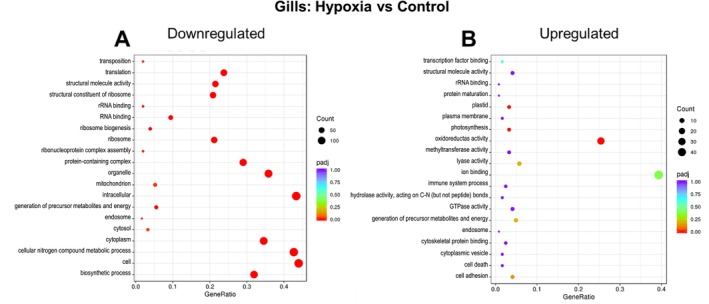
GO pathway enrichment in the gills of hypoxia *L. capensis* relative to the normoxic control. Colour indicates the significance (*p*_adj_) and size of the symbols—the number of DEGs found in the respective pathway. The *X*‐axis shows Gene Ratio for each pathway, depicting the ratio of differentially expressed genes to all genes for this GO term. A ‐ upregulated DEGs, B ‐ downregulated DEGs.

Exposure to hypoxia in the gills of *L. capensis* resulted in the significant downregulation of numerous GO pathways (*p*adj < 0.05), particularly those linked to protein metabolism and cellular biosynthesis (Figure 4A). The regulation of mobile genetic elements (transposition) was also notably enriched among downregulated DEGs. Conversely, only three GO pathways—associated with endosymbiont metabolism (plastid, photosynthesis, and oxidoreductase activity)—were significantly enriched among the upregulated genes (*p*adj < 0.05) (Figure 4B). The DEGs associated with plastid and photosynthesis GO pathways included several subunits of ribulose‐1,5‐bisphosphate carboxylase/oxygenase (RuBisCO), a key enzyme in carbon fixation via the Calvin–Benson–Bassham cycle.

**FIGURE 5:**
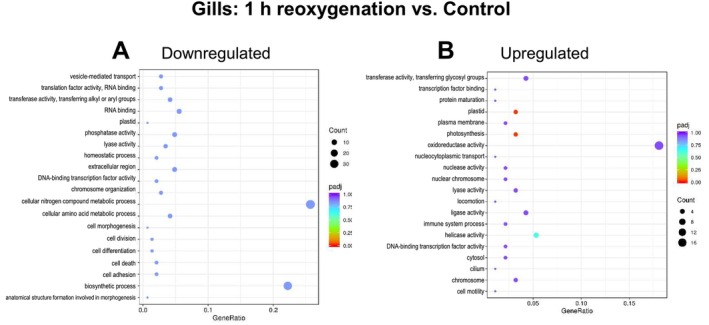
GO pathway enrichment in the gills of *L. capensis* after 1 h of reoxygenation relative to the normoxic control. Colour indicates the significance (*p*_adj_) and size of the symbols—the number of DEGs found in the respective pathway. The *X*‐axis shows Gene Ratio for each pathway, depicting the ratio of differentially expressed genes to all genes for this GO term. A ‐ upregulated DEGs, B ‐ downregulated DEGs.

**FIGURE 6:**
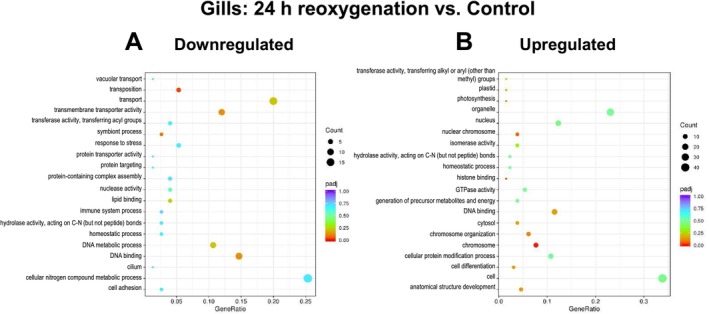
GO pathway enrichment in the gills of *L. capensis* after 24 h of reoxygenation relative to the normoxic control. Colour indicates the significance (*p*_adj_) and size of the symbols—the number of DEGs found in the respective pathway. The *X*‐axis shows Gene Ratio for each pathway, depicting the ratio of differentially expressed genes to all genes for this GO term. A ‐ upregulated DEGs, B ‐ downregulated DEGs.

After 1 h of reoxygenation, no significantly downregulated GO pathways were detected in the gills of *L. capensis* compared to the normoxic state (Figure 5A,C). However, two GO pathways associated with endosymbiont metabolism (plastid, and photosynthesis both involving RuBisCO subunits) were significantly enriched among upregulated genes (*p*adj < 0.05) (Figure 5B). After 24 h of reoxygenation, the transposition GO pathway was significantly downregulated (*p*_adj_ < 0.05) relative to the normoxic state in the gills of *L. capensis* (Figure 6A). Two GO pathways (chromosome and histone binding) were significantly enriched (*p*_adj_ < 0.05) among the upregulated genes in *L. capensis* gills after 24 h of recovery (Figure 6B).

In the digestive gland tissue, no significantly enriched GO pathways were identified among the downregulated transcripts under hypoxia (Figure S3A). However, three GO pathways related to the extracellular region, extracellular space and enzyme regulator activity were significantly upregulated in the digestive gland of hypoxia‐exposed *L. capensis* compared to the normoxic control (*p*_adj_ < 0.05) (Figure S3B). After 24 h of post‐hypoxia recovery, no significantly enriched down‐ or upregulated GO pathways were found in the digestive gland compared to normoxic controls (*p*_adj_ > 0.05) (Figure S4). The lysosome pathway was the only one found to be enriched in the digestive gland after 24 h of recovery compared to the hypoxia state, and it was downregulated (*p*_adj_ < 0.05; Figure S5).

### 3.6 Reactome Metabolic Pathway Enrichment Analysis

Reactome analysis of eukaryotic DEGs revealed significant downregulation (FDR < 0.05) of 42 metabolic pathways in the hypoxic gills of *L. capensis* (Table S5). Because human homologues were used for pathway inference, these results serve as proxies for essential cellular and molecular interactions rather than direct one‐to‐one matches with bivalve physiology. The suppressed pathways were mainly related to protein and RNA metabolism, cellular stress responses (e.g., amino acid deficiency), metabolism, nervous system development, and immune defence. Eighteen pathways showed particularly strong enrichment, with > 50% of their genes downregulated under hypoxia. These included nonsense‐mediated decay (NMD) of mRNA, ribosome biogenesis and regulation, protein translation, viral–host interactions, and selenoamino acid metabolism. Other significantly downregulated pathways (< 50% of genes affected) were also linked to ribosome biogenesis, protein translation, viral–host interactions, cellular stress, starvation, metabolism, and neural development (Table S5).

An additional six pathways showed moderate evidence (0.05 < FDR < 0.1) for downregulation in the gills of the hypoxia‐exposed *L. capensis* . Interestingly, three of these pathways were related to mitochondrial OXPHOS, including R‐HAS‐163200 (respiratory electron transport, ATP synthesis by chemiosmotic coupling, and heat production by uncoupling proteins), and R‐HSA‐6799198 (Complex I biogenesis) pathways, with 9–17 downregulated DEGs (corresponding to 12%–16% of all genes in the respective pathway) (Table S5). Reactome analysis identified a single pathway that showed moderate evidence of upregulation in the gills of hypoxia‐exposed *L. capensis* (the citric acid (TCA) cycle and respiratory electron transport, FDR = 0.06) (Table S5). Notably, there was no evidence for alteration of glycolysis (Reactome pathway R‐HSA‐70171) in the gills or the digestive gland of the clams.

Metabolic pathways in the digestive gland of *L. capensis* were less affected by hypoxia exposure than those in the gills (Table S6). No pathways were significantly enriched for downregulated genes in the digestive gland of hypoxia‐exposed clams (FDR > 0.1), and only three pathways related to membrane receptor‐mediated signalling and immune function showed moderate evidence of upregulation (0.05 < FDR < 0.1) (Table S6).

Despite the large number of DEGs found in the gills of *L. capensis* during post‐hypoxia recovery relative to both hypoxia and the normoxic control, no overrepresentation of these genes in specific metabolic pathways was identified by Reactome (FDR > 0.1). Similarly, the DEGs up‐ and downregulated in the digestive gland after 24 h of recovery relative to the control were not significantly overrepresented in any of the Reactome pathways (FDR > 0.1). However, in the digestive gland the downregulated DEGs after 24 h of reoxygenation relative to hypoxia (FDR < 0.1) were overrepresented in nine pathways associated with extracellular matrix maintenance and immune function (Table S6). No pathway overrepresentation of the upregulated DEGs after 24 h of recovery relative to hypoxia was identified by the Reactome in the digestive gland (FDR > 0.1).

### 3.7 Metabolite Profiles

**FIGURE 7:**
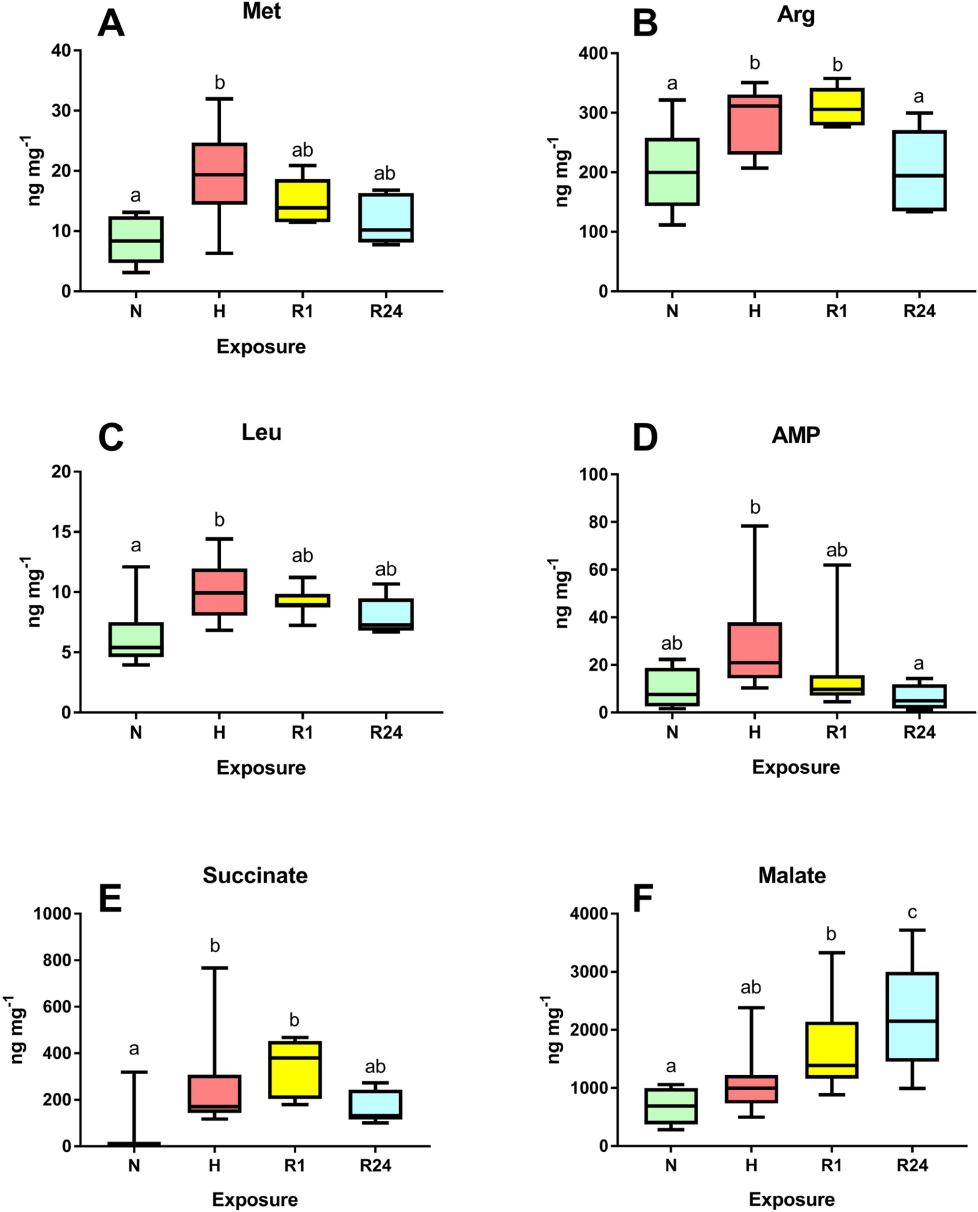
Effect of different oxygen conditions on concentrations of selected metabolites in the gills of *L. capensis* . Only metabolites that show significant effects of oxygen conditions (*p* < 0.05) are shown. Different letters indicate significant differences between the values for the respective exposure groups (*p* < 0.05). Conditions: N—normoxia, H—hypoxia, R1—1 h reoxygenation, R24—24 h reoxygenation. *N* = 5–11 per group. A ‐ methionine, B ‐ arginine, C ‐ leucine, D ‐ AMP, E ‐ succinate, F ‐ malate.

Of the 35 metabolites quantified in the gills and digestive gland of *L. capensis* , six showed significant effects of the oxygen regime in the gills (*p* < 0.05). Three amino acids (Met, Arg and Leu) accumulated during hypoxia exposure in the gill and gradually decreased to the baseline levels during reoxygenation (Figure 7A–C). Similarly, AMP accumulated in *L. capensis* gills during hypoxia and returned to the normoxic baseline after 1 h of reoxygenation (Figure 7D). Succinate accumulated in the gills of *L. capensis* during hypoxia and continued to increase during the 1st hour of reoxygenation, declining only after 24 h of post‐hypoxia recovery (Figure 7E). Malate concentration in the gills moderately increased during hypoxia and continued to rise throughout the 1–24 h post‐hypoxia recovery period (Figure 7F).

**FIGURE 8:**
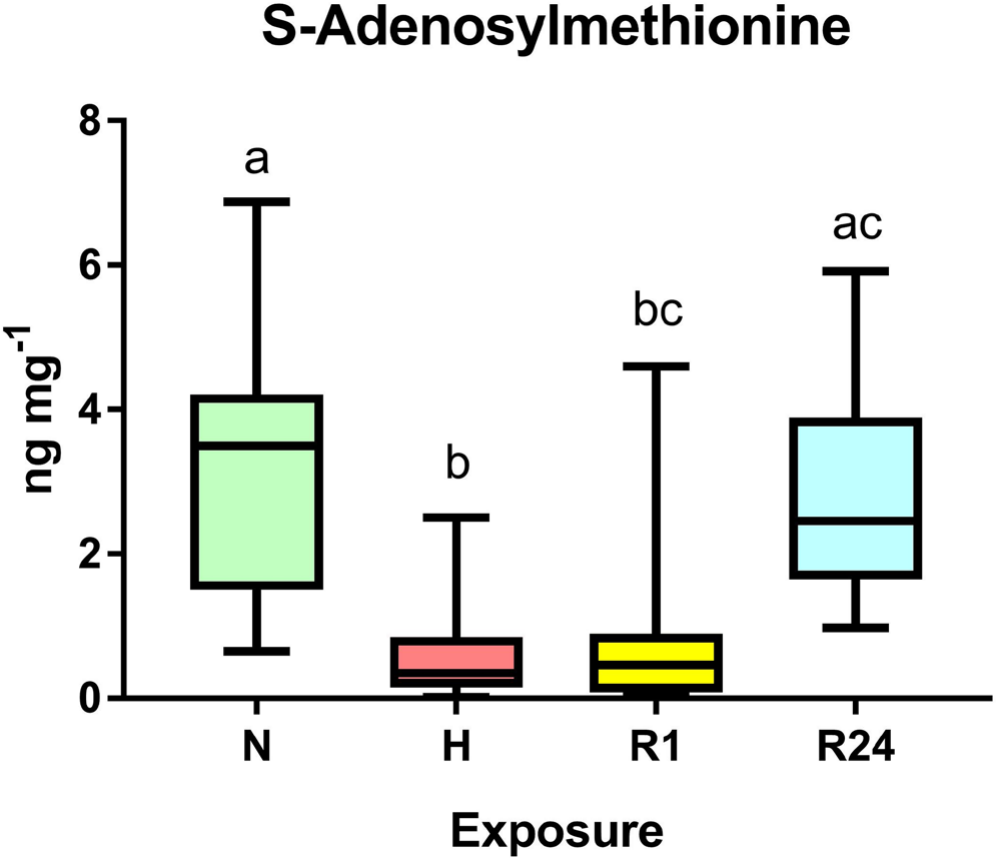
Effect of different oxygen conditions on concentrations of SAM in the digestive gland of *L. capensis* . Different letters indicate significant differences between the values for the respective exposure groups (*p* < 0.05). Conditions: N—normoxia, H—hypoxia, R1—1 h reoxygenation, R24—24 h reoxygenation. *N* = 7–11 per group.

In the digestive gland, only S‐adenosylmethionine (SAM) content showed significant differences between exposure groups (*p* < 0.05). SAM concentrations in the digestive gland decreased in hypoxia and were partially restored after 24 h of reoxygenation (Figure 8).

**FIGURE 9:**
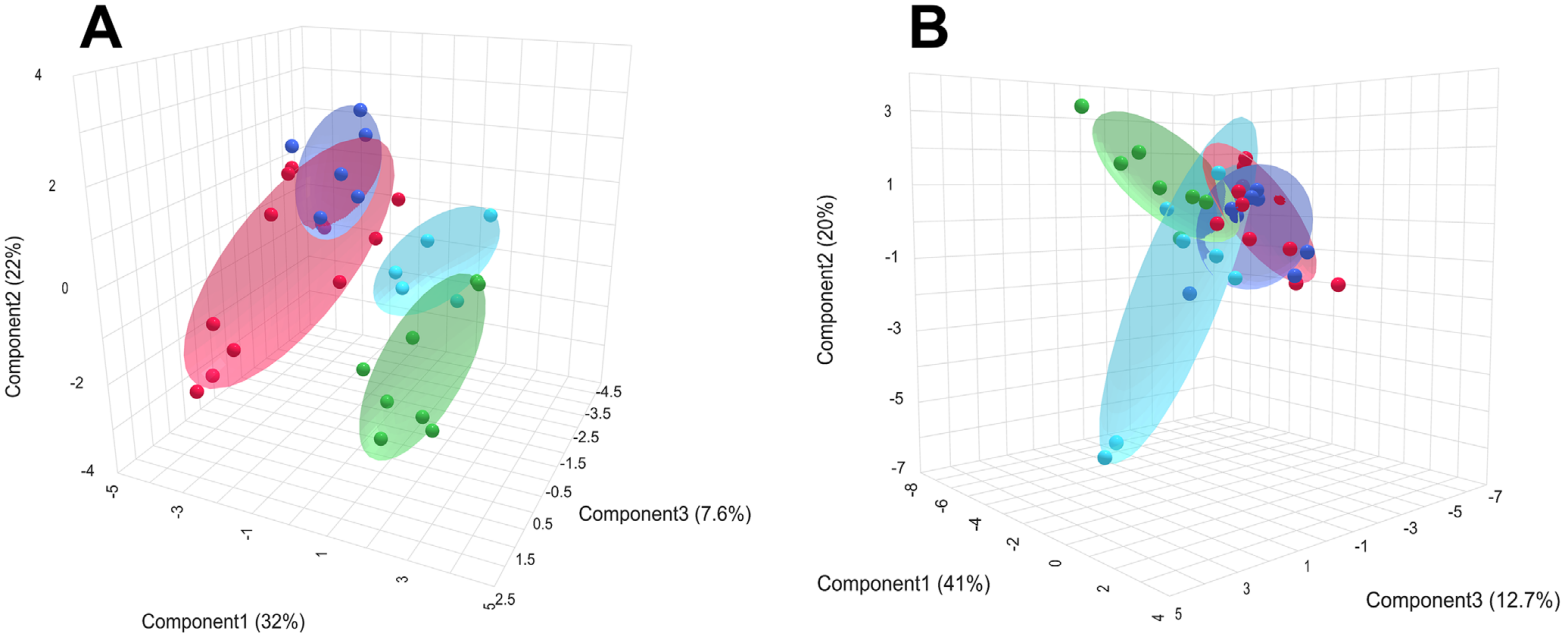
sPLS‐DA 3D plots of the metabolite profiles of *L. capensis* gills (A) and the digestive gland (B) exposed to different oxygen conditions. Individual samples (shown by dots) are plotted in the space of three first principal components (PC). Numbers in brackets show the percentage of the data variance explained by a certain PC. Shaded ellipsoids show 95% confidence intervals.

The sPLS‐DA analysis revealed a clear separation of metabolite profiles in the gills between clams from all exposure groups and the normoxic control (Figure 9A). Metabolite profiles at early recovery (1 h) closely resembled those of the hypoxia‐exposed groups, but after 24 h of recovery, they shifted towards the normoxic control profiles. In contrast, there was considerable overlap in metabolite profiles among the different oxygen exposure groups in the digestive gland, suggesting a less distinct metabolic response to oxygen variations in this tissue (Figure 9B).

Metabolome‐based pathway enrichment analysis did not reveal any significantly enriched metabolic pathways between the normoxic and hypoxic gills of *L. capensis* , or between the normoxic gills and those after 24 h of reoxygenation (FDR > 0.1). However, significant alterations were observed in the TCA cycle (FDR < 0.05) as well as in pyruvate metabolism, butanoate metabolism, and cysteine and methionine metabolism (FDR < 0.1) in the gills during the first hour of post‐hypoxia recovery (Table S7). In the digestive gland, no significantly enriched metabolic pathways were detected in either hypoxia or post‐hypoxia recovery compared to the normoxic control (FDR > 0.1).

## 4 Discussion

### 4.1 Hypoxia Response in *L. capensis* Gill Involves Metabolic and Immune Suppression

The OMZ bivalve *L. capensis* exhibited a significant transcriptional response to hypoxia, with marked differences between the gills and the digestive gland. The gill transcriptome was considerably more responsive, showing 9003 DEGs between normoxic and hypoxic conditions, compared to 2697 DEGs in the digestive gland. However, only a small portion (~14%–20%) of these DEGs could be assigned a putative function based on sequence homology with available gene and protein libraries, likely reflecting the high divergence of this deep‐sea lucinid genome (Shin et al. 2024; Taylor et al. 2016, 2011). When focusing on genes with identified functions, the hypoxia‐induced transcriptomic shift remained more pronounced in the gills (1234 DEGs) than in the digestive gland (549 DEGs). While the smaller sample size for the digestive gland may contribute to this difference, metabolomic data also showed a lower level of hypoxia‐induced change, suggesting the digestive gland is less sensitive to oxygen fluctuations than the gills. The gills serve as the primary organ for gas exchange and feeding in bivalves (Carroll and Catapane 2007; Morton 1960) and their function is highly sensitive to oxygen availability. Under severe hypoxia, shell closure stops oxygen and food intake, and as the limited oxygen within the shell is rapidly depleted, the bivalves transition to anaerobic metabolism (Brinkhoff et al. 1983; Ortmann and Grieshaber 2003).

Anaerobic metabolism presents challenges, including energy deficiency from lower ATP yield, accumulation of toxic byproducts, and metabolic acidosis (Brinkhoff et al. 1983; Hochachka and Mommsen 1983; Hochachka and Mustafa 1972; Pörtner et al. 1984). A key survival strategy in anoxia‐tolerant animals is suppressing metabolic rate, reducing both ATP production and consumption (Hochachka et al. 1996; Hochachka and Guppy 1987). Protein turnover is one of the most energy‐demanding cellular processes (Li et al. 2014; Sokolova 2021), and our study shows that downregulation of protein synthesis during hypoxia is a key energy‐saving mechanism in the gills of *L. capensis* . GO enrichment and Reactome analyses revealed coordinated downregulation of protein transcription, translation, and transport, along with suppressed amino acid metabolism, under hypoxic conditions. Notably, the cellular response to starvation (e.g., Reactome R‐HSA‐9711097), typically activated by amino acid deprivation, was also downregulated, suggesting an adaptive strategy that prioritises essential survival functions over costly responses to amino acid depletion. Similarly, energy‐intensive processes related to neural development (e.g., SLIT and ROBO signalling, axon guidance) were downregulated, likely reflecting either an energy‐saving mechanism or a secondary effect of reduced protein synthesis.

Although pathway enrichment analyses did not identify proteolysis or protein catabolic processes among the significantly enriched GO terms in *L. capensis* gills, a substantial proportion of DEGs—62 downregulated and 25 upregulated (12% and 11% of the total DEG pools, respectively)—were associated with proteolysis. In the digestive gland, the transcriptomic response to hypoxia was weaker, with fewer DEGs and significantly enriched pathways compared to the gill. However, protein synthesis regulation (18 downregulated DEGs, 11% of the altered transcriptome) and proteolysis (11 upregulated and 38 downregulated DEGs, 6% and 10% of the altered transcriptome, respectively) were prominent. Notably, proteolysis‐related genes were the largest or second‐largest functional category of DEGs in both tissues. The lack of significant GO term enrichment despite a high abundance of proteolysis‐related DEGs likely reflects their distribution across various sub‐processes, diluting the enrichment signal. Nonetheless, the prominence of proteolysis‐related DEGs is biologically significant in hypoxia, where protein synthesis shutdown necessitates both the extended function of existing proteins (Hand and Hardewig 1996), and the efficient removal of damaged proteins (Agrawal et al. 2017; Fawcett et al. 2015). Thus, proteolytic pathway regulation is essential for balancing protein turnover during metabolic suppression in hypoxic tissues of *L. capensis* , even if not captured in pathway enrichment analyses.

Intriguingly, a substantial proportion of downregulated genes in the gills of *L. capensis* were associated with immune functions (62 DEGs, 8%). In contrast, this immune suppression was not evident in the digestive gland, where only 7 immune‐related genes (4%) were downregulated, while 62 (16%) were upregulated. Reactome analysis further indicated enrichment of these downregulated immune genes in pathways linked to viral‐host interactions in the gills. This gill‐specific transcriptional suppression of immune pathways likely represents an energy‐conserving adaptation to hypoxic stress, rather than a response to an active immune challenge. Similar patterns of immune gene modulation under hypoxia have been observed in other bivalves, including the shallow‐water mytilids *Mytilus edulis* and *M. chilensis* , as well as the arcid clam *Tegillarca granosa* (Cheng et al. 2024; Montúfar‐Romero et al. 2024; Wu, Kong, et al. 2024), suggesting that hypoxia‐driven immunomodulation may be a conserved response across Bivalvia.

The downregulation of transposition‐related pathways and transposable element (TE) dynamics (58 DEGs, 7%) in the gills of *L. capensis* suggests a strategy to maintain genomic integrity under environmental stress. TEs make up a large share of genomes (3%–50%) and can be mobilised by stressors such as temperature shifts, UV, oxidative stress, or nutrient limitation (Capy et al. 2000; de Oliveira et al. 2021; Pappalardo et al. 2021). While TE activation may promote adaptation by increasing genetic variability and stress‐responsive networks, their regulation is stress‐specific, with some families repressed and others activated (de Oliveira et al. 2021). Studies in mammalian cells show hypoxia suppresses TE transcription, while derepression triggers immune hyperactivation, DNA damage, and cell death (Park et al. 2023). Thus, TE suppression in *L. capensis* gills may stabilise the genome, prevent harmful immune activation, and conserve energy. Coordinated downregulation of TE and immune pathways could also protect endosymbionts while prioritising essential metabolic processes over immunity under oxygen limitation. In sum, this suppression reflects an adaptive strategy to optimise energy use, preserve symbiotic associations, and maintain genomic stability in an unstable, hypoxic environment.

As expected, oxidative phosphorylation (OXPHOS) was suppressed in the hypoxic gills of *L. capensis* , involving downregulation of respiratory electron transport, ATP synthesis, and Complex I biogenesis. Because Complex I is a major source of reactive oxygen species (ROS; Brondani et al. 2023; Chouchani et al. 2016; Sokolov et al. 2021), its suppression may limit oxidative stress, especially during reoxygenation. Notably, we detected no transcriptional upregulation of antioxidant pathways in the gills or digestive gland, either during hypoxia or reoxygenation. Instead, selenoamino acid metabolism, including selenocysteine synthesis, was downregulated. Selenocysteine, incorporated into selenoproteins such as glutathione peroxidases and thioredoxin reductases, supports antioxidant defence and redox regulation (Steinbrenner et al. 2016). The reduced transcription of these pathways suggests lower demand for antioxidant activity in hypoxia‐exposed clams. This is consistent with reports that bivalve mitochondria do not generate significant ROS during reoxygenation and demonstrate minimal reverse electron transport through Complex I (Adzigbli et al. 2024, 2022; Sokolov et al. 2021; Steffen et al. 2021, 2023). Comparative studies further show that basal levels and inducibility of antioxidant enzymes do not correlate with thermal tolerance across bivalves (Dowd and Somero 2023; Falfushynska et al. 2016). Together, these findings suggest that *L. capensis* and other stress‐tolerant bivalves rely on regulating or tolerating ROS production rather than boosting antioxidant defences—a strategy that also conserves energy by reducing the need for de novo synthesis of antioxidant proteins during hypoxia.

The gills of *L. capensis* harbour sulfur‐oxidising symbionts (*Candidatus Thiodiazotropha*) that detoxify H_2_S and support the host by fixing CO_2_ and assimilating ammonium under hypoxia (Amorim et al. 2022; Cary and Felbeck 1989; Yuen et al. 2019). In this study, we observed upregulation of prokaryotic transcripts associated with sulfur metabolism, CO_2_ fixation, electron transport, and protein synthesis—potentially reflecting enhanced symbiont activity related to detoxification and energy production during hypoxia. However, interpretation of the bacterial signal is constrained by the use of poly‐A enrichment, which biases against non‐polyadenylated microbial RNA, and the taxonomic resolution of the data does not allow for definitive attribution of transcripts to *Candidatus Thiodiazotropha*. Despite these limitations, the functional signatures detected in the gills are consistent with the established roles of chemosynthetic symbionts in lucinid clams and provide preliminary insight into their transcriptional responses to low oxygen conditions.

### 4.2 Hypoxic Digestive Gland of *L. capensis* Upregulates Structural Maintenance

In comparison to the gill, the transcriptomic response of the digestive gland was notably muted, with only 167 downregulated and 382 upregulated differentially expressed genes (DEGs). This attenuated response may be attributed to the digestive gland's distinct physiological role. While the gills are directly involved in oxygen uptake, the digestive gland primarily functions in digestion and nutrient storage, activities not immediately related to gas exchange (Lobo‐da‐Cunha 2019). Moreover, as an internal organ, the digestive gland may encounter hypoxic conditions in a delayed or buffered manner compared to the gills. Transcriptomic analyses in the Pacific oyster, *Crassostrea gigas* , revealed differential expression of prolyl hydroxylase domain‐containing protein 2 (PHD2) isoforms between the gills and the digestive gland, with the gills expressing a more oxygen‐sensitive, strongly inducible, and active isoform (Meng et al. 2022). These findings suggest that the reduced sensitivity of the digestive gland transcriptome to hypoxia reflects both the functional specialisation and molecular composition of the oxygen‐sensing pathways in this organ, which make it less responsive to fluctuations in oxygen availability.

In the digestive gland transcriptome of *L. capensis* exposed to 36 h of severe hypoxia, no significantly downregulated pathways were observed. However, two pathways related to extracellular matrix (ECM) formation and tissue maintenance, mediated by fibroblast growth factor receptor (FGFR) 2 activation, were upregulated. While the role of FGFR in molluscs is not well understood, in mammals, FGFR signalling regulates cell proliferation, differentiation, and tissue repair (Xie et al. 2020). The upregulation of FGFR signalling in *L. capensis* likely reflects an increased investment in tissue maintenance and repair under hypoxia. This hypothesis is supported by the analysis of manually annotated genes showing upregulation of genes involved in ECM formation and maintenance, representing 8% of the upregulated transcriptome (32 DEGs), including those regulating collagen metabolism, proteoglycan synthesis, as well as cellular adhesion (16 DEGs, 4% of the upregulated transcriptome). These findings suggest a focus on preserving the structural integrity of the digestive gland and its epithelium. Additionally, the observed upregulation of immune‐related genes (62 DEGs, 16% of the upregulated transcriptome), particularly those involved in the trafficking and processing of endosomal Toll‐like receptors (TLRs) (Reactome pathway R‐HSA‐1679131), further supports this interpretation. TLRs are critical in tissue repair and regeneration, as they respond to pathogen‐associated molecular patterns (PAMPs) released by the microbiome or damage‐associated molecular patterns (DAMPs) from dying cells, triggering tissue recovery processes (Ioannou and Voulgarelis 2010). Studies on the mud crab *Scylla paramamosain* have demonstrated a similar role for FGFR signalling in regulating the TLR pathway (Li et al. 2022), suggesting that this function is conserved across invertebrates and vertebrates. These findings suggest that the hypoxic response in the digestive gland of *L. capensis* prioritises tissue integrity through ECM and epithelial maintenance, supported by the absence of significant downregulation of protein synthesis in this organ.

### 4.3 Metabolome Stability During Oxygen Fluctuations in *L. capensis*

Despite substantial transcriptomic reorganisation, the tissue metabolome of both the gill and digestive gland of *L. capensis* remained remarkably stable during oxygen fluctuations. Of the 35 quantified metabolites in the gill, only five showed significant changes under hypoxia, and three in response to reoxygenation, compared to the normoxic baseline. Succinate accumulation in hypoxic gills, a typical response in marine bivalves, indicates a shift to mitochondrial anaerobiosis (Pörtner et al. 1984; Tielens et al. 2002). Notably, no alanine accumulation was observed, suggesting that cytosolic anaerobiosis is either bypassed or transient in this hypoxia‐adapted species. Elevated AMP levels reflect an imbalance between ATP breakdown and resynthesis, reducing cellular energy charge, a trend quickly reversed upon reoxygenation, along with a malate overshoot, indicating TCA reactivation. Three amino acids (Met, Leu, Arg) accumulated under hypoxia, returning to baseline after reoxygenation (Met, Leu at 1 h; Arg at 24 h). Arginine accumulation likely reflects phosphagen breakdown for anaerobic ATP production (Livingstone 1991), while Met and Leu accumulation might be due to a hypoxia‐induced shutdown of protein synthesis.

In the digestive gland, the only metabolite showing a significant response to hypoxia was a marked (~5.4‐fold) decrease in S‐adenosylmethionine (SAM) content. This decline points to reduced methylation capacity, with possible consequences for DNA and protein methylation (Loenen 2006). Because SAM is central to numerous cellular processes, it remains difficult to pinpoint which functions in the digestive gland of *L. capensis* are most affected. Importantly, SAM levels recovered rapidly upon reoxygenation, returning to normoxic values within 24 h, suggesting that any downstream effects are likely transient. No evidence of increased anaerobic ATP production was observed in the digestive gland, as neither succinate nor alanine accumulated. This aligns with transcriptomic data showing low oxygen sensitivity in this tissue and implies that its ATP requirements during 36 h of hypoxia are sustained either through residual aerobic metabolism or via hemolymph‐mediated supply of energy and nutrients from symbiont‐rich tissues, such as the gill (Frizzo et al. 2021).

When comparing the hypoxia‐induced metabolome shifts of *L. capensis* with other marine bivalves, including hypoxia‐tolerant intertidal species, *L. capensis* demonstrates remarkable metabolic resilience. In *M. edulis* , for instance, 24 h of severe hypoxia caused significant shifts in 19 out of 20 amino acids in both the gills and whole body, with most amino acids accumulating, while others, such as aspartate (utilised in anaerobic alanine production), were depleted (Haider et al. 2020; Wu, Sokolov, et al. 2024). Similarly, in the Pacific oyster *Crassostrea gigas* , hypoxia led to the accumulation or depletion of several amino acids—7 out of 20 after 24 h and 15 out of 20 after 6 days (Haider et al. 2020). Furthermore, *M. edulis* experienced hypoxia‐induced changes in metabolic pathways related to energy, carbohydrate, amino acid, and cofactor metabolism (Wu, Sokolov, et al. 2024). In *C. gigas* , intermittent hypoxia affected multiple key pathways, including alanine, aspartate, and glutamate metabolism, arginine biosynthesis, metabolism of aromatic amino acids, and the TCA cycle (Bruhns et al. 2023). Even mild hypoxia (2 mg L^−1^ O_2_) in the pearl oyster *Pinctada fucata martensii* triggered a substantial reorganisation of 25 metabolic pathways, including metabolism of tRNA, amino acids, glycerophospholipids, and ABC transporter activity (Yang et al. 2023). In contrast, the metabolome of *L. capensis* in both the gill and digestive gland remained stable during hypoxia and reoxygenation, underscoring its exceptional resilience to oxygen fluctuations.

### 4.4 Recovery: A Return to Baseline or a Distinct Metabolic State?

Pathway enrichment analyses revealed that immunosuppression initiated during hypoxic exposure in the gills of *L. capensis* intensified during the first hour of reoxygenation, as shown by the suppression of multiple immune‐related pathways. Additionally, inflammation signalling pathways were significantly suppressed during the first hour of reoxygenation compared to the hypoxic state. After 24 h of reoxygenation, no immunity‐related pathways were significantly enriched relative to the normoxic baseline or the hypoxic state, despite the identification of 29 downregulated and 11 upregulated immune‐related DEGs compared to the baseline, indicating a gradual return to the normoxic baseline state.

Differential regulation of gill proteolysis during recovery indicates a major shift in cellular priorities upon reoxygenation. Within the first hour, 49 proteolysis‐related DEGs were downregulated (12% of the downregulated pool) and 27 were upregulated (9% of the upregulated pool) relative to normoxia, suggesting selective protein degradation early in recovery. After 24 h, this trend persisted with 27 downregulated and 23 upregulated DEGs (8% and 9% of their respective pools). Despite the significant representation of proteolysis‐related DEGs in the recovering gill transcriptome, the absence of specific enriched pathways indicates that these genes are widely distributed among various pathways and subprocesses. By contrast, relatively few protein synthesis genes were differentially expressed during recovery—23 and 20 downregulated DEGs after 1 and 24 h, respectively, with 12 upregulated at both phases—compared to hypoxia, where 150 were downregulated and 12 upregulated. Notably, two GO pathways, chromosome binding and histone binding, were enriched among upregulated genes after 24 h, suggesting enhanced transcriptional regulation. Together, these results indicate a rapid restoration of proteosynthetic activity as gills shift from hypoxia to reoxygenation.

Overall, reoxygenation leads to reactivation of essential cellular processes, with protein synthesis restored rapidly and other functions, such as protein degradation and immune activity, recovering more gradually. The number of downregulated DEGs decreased steadily from hypoxia (819) to 1 h of recovery (409) and further to 24 h (261), reflecting the progressive release of processes suppressed during hypoxia as part of metabolic depression. At the same time, recovery was marked by substantial transcriptional upregulation, with 289 and 328 DEGs exceeding the normoxic baseline after 1 and 24 h, respectively. These responses are not simple continuations of hypoxic regulation but instead reflect distinct functional shifts, with distinct categories of DEGs overrepresented during recovery compared to hypoxia. Metabolomic evidence of an overshoot in TCA cycle activity supports the idea that recovery involves active regulation of metabolism rather than a passive return to baseline. In hypoxia‐tolerant organisms, recovery often coincides with elevated oxygen consumption, the so‐called “oxygen debt” (Lewis et al. 2007; Van den Thillart and Verbeek 1991; Vismann and Hagerman 1996). Our findings indicate that this metabolic surge might be driven by broad stimulation of cellular processes across multiple networks in the gills, particularly those related to proteome homeostasis and immune defence, during recovery.

Recovery in the digestive gland was less pronounced, consistent with the weaker transcriptomic response of this tissue to hypoxia compared to the gills. DEG numbers were similar between hypoxia (167 downregulated, 382 upregulated) and 24 h recovery (144 downregulated, 384 upregulated). The only significantly enriched pathway among downregulated DEGs during recovery was the lysosome pathway, suggesting suppression of intracellular digestion—likely an energy‐saving strategy to minimise specific dynamic action (Goodrich et al. 2024). Overall, the digestive gland showed notable transcriptomic and metabolic stability across oxygen fluctuations, underscoring its resilience and lower sensitivity to hypoxia relative to the more responsive gills.

## 5 Conclusions

This study provides the first comprehensive insights into the metabolic regulation of the OMZ‐adapted bivalve *L. capensis* in response to fluctuating oxygen conditions. The data reveal pronounced organ‐specific specialisation, with the gills exhibiting strong transcriptional responses to oxygen variability, in stark contrast to the relative transcriptional stability observed in the digestive gland.

Under hypoxia, *L. capensis* gills showed a coordinated suppression of major metabolic processes, including protein synthesis, transposable element activity, and immune function—reflecting a tightly controlled energy conservation strategy that also safeguards symbiont stability and genomic integrity. At the same time, signs of endosymbiont metabolic activation were evident, consistent with its central role in host energy provision and sulfide detoxification during oxygen limitation (Amorim et al. 2022; Cary and Felbeck 1989; Yuen et al. 2019). Reoxygenation induced an active and asymmetric recovery trajectory in the gill, characterised by a rapid restoration of protein synthesis and a more gradual normalisation of protein degradation and immune‐related processes. The overshoot observed in TCA cycle intermediates, alongside the derepression of pathways previously downregulated during hypoxia, indicates that reoxygenation involves active metabolic reprogramming rather than a simple return to baseline. In contrast, the digestive gland showed far fewer transcriptional and metabolomic changes during oxygen fluctuations, with selective pathway upregulation pointing to investment into structural maintenance under hypoxia.

Together, these findings highlight the organ‐level metabolic specialisation that underpins the resilience of *L. capensis* to OMZ dynamics. This work provides essential groundwork for understanding the molecular strategies enabling benthic invertebrates to persist in increasingly oxygen‐depleted marine environments.

## Author Contributions

**Inna M. Sokolova:** conceptualization, formal analysis, data curation, visualization, supervision, writing – original draft, writing – review and editing. **Eugene P. Sokolov:** data curation, formal analysis, writing – review and editing. **Helen Piontkivska:** methodology, validation, formal analysis, data curation, funding acquisition, writing – review and editing. **Stefan Timm:** investigation, methodology, validation, resources, writing – review and editing. **Katherine Amorim:** investigation, writing – review and editing. **Michael L. Zettler:** conceptualization, supervision, project administration, funding acquisition, writing – review and editing.

## Funding

This work was supported by Bundesministerium für Bildung und Forschung (03V01279); Hochschul‐Bau‐Förderungsgesetz (GZ INST 264/125‐1FUGG); Mare Balticum Fellowship, Universität Rostock and National Commission on Research, Science and Technology (RPIV00812019).

## Conflicts of Interest

The authors declare no conflicts of interest.

## Supporting information


**Table S1:** List of the functional categories used for classification of eukaryotic and prokaryotic DEGs in the manually curated database of *L. capensis* .
**Table S2:** Relative frequency of functional gene categories (as listed in Table S1) among differentially expressed eukaryotic genes (DEGs) identified in the gill tissues of *L. capensis* exposed to varying oxygen conditions.
**Table S3:** Relative frequency of functional gene categories (as listed in Table S1) among differentially expressed prokaryotic genes (DEGs) identified in the gill tissues of *L. capensis* exposed to varying oxygen conditions.
**Table S5:** Results of the Reactome analysis of the gill transcriptome of *L. capensis* , displaying only significantly up‐ or downregulated pathways (FDR < 0.1). No significantly enriched pathways were identified in the following comparisons: R24 (24 h of reoxygenation) versus normoxia (N), R1 (1 h of reoxygenation) versus hypoxia (H), and R24 versus hypoxia. Additionally, no significantly downregulated pathways were found in the R1 versus N comparison. “# Genes found” refers to the number of identified DEGs, while “# Genes total” refers to the total number of genes in the respective pathway. Rxn—reactions. For the sake of completeness, we report all pathways identified as significantly enriched by Reactome analysis. Pathways that are specific to vertebrates and are unlikely to be relevant in molluscs are indicated in italics.
**Table S6:** Results of the Reactome analysis of the digestive gland transcriptome of *L. capensis* , displaying only significantly up‐ or downregulated pathways (FDR < 0.1). No significantly downregulated pathways were identified in the following comparisons: hypoxia (H) versus normoxia (N), R24 (24 h of reoxygenation) versus N. No significantly downregulated pathways were identified in the following comparisons: R24 versus N and R24 versus A. “# Genes found” refers to the number of identified DEGs, while “# Genes total” refers to the total number of genes in the respective pathway. Rxn—reactions.
**Table S7:** Significantly enriched pathways identified through MetaboAnalyst analysis of *L. capensis* gill metabolome profiles under normoxic conditions versus 1 h of reoxygenation. Only pathways with a False Discovery Rate (FDR) < 0.1 are included. “Total Compounds” refers to the total number of compounds associated with each pathway, while “Hits” indicates the number of metabolites detected in *L. capensis* gills that are linked to each pathway.
**Figure S1:** GO pathway enrichment in the gills of *L. capensis* after 1 h of reoxygenation relative to the hypoxic state. Colour indicates the significance (*p*_adj_) and size of the symbols—the number of DEGs found in the respective pathway. The *X*‐axis shows Gene Ratio for each pathway, depicting the ratio of differentially expressed genes to all genes for this GO term.
**Figure S2:** GO pathway enrichment in the gills of *L. capensis* after 24 h of reoxygenation relative to the hypoxic state. Colour indicates the significance (*p*_adj_) and size of the symbols—the number of DEGs found in the respective pathway. The *X*‐axis shows Gene Ratio for each pathway, depicting the ratio of differentially expressed genes to all genes for this GO term.
**Figure S3:** GO pathway enrichment in the hypoxic digestive gland of *L. capensis* relative to the normoxic control. Colour indicates the significance (*p*_adj_) and size of the symbols—the number of DEGs found in the respective pathway. The *X*‐axis shows Gene Ratio for each pathway, depicting the ratio of differentially expressed genes to all genes for this GO term.
**Figure S4:** GO pathway enrichment in the digestive gland of *L. capensis* after 24 h of reoxygenation relative to the normoxic control. Colour indicates the significance (*p*_adj_) and size of the symbols—the number of DEGs found in the respective pathway. The *X*‐axis shows Gene Ratio for each pathway, depicting the ratio of differentially expressed genes to all genes for this GO term.
**Figure S5:** GO pathway enrichment in the digestive gland of *L. capensis* after 24 h of reoxygenation relative to the hypoxic state. Colour indicates the significance (*p*_adj_) and size of the symbols—the number of DEGs found in the respective pathway. The *X*‐axis shows Gene Ratio for each pathway, depicting the ratio of differentially expressed genes to all genes for this GO term.

## Data Availability

The transcriptomic data have been deposited in NCBI GenBank Sequence Read Archive under BioProject accession number PRJNA1244758. The metabolomic dataset and the dataset related to the transcriptomic analyses are publicly available on Zenodo at https://doi.org/10.5281/zenodo.15268761 and https://doi.org/10.5281/zenodo.16367847, respectively, under the Creative Commons Attribution Share Alike 4.0 International Licence.
