# Supplementary material for: Unravelling Hypoxia Tolerance: Transcriptomic and Metabolic Insights From Lucinoma capensis in an Oxygen Minimum Zone
Source: Mol Ecol. 2025 Dec 1;34(24):e70194. doi: 10.1111/mec.70194 (PMC12717985; doi:10.1111/mec.70194)
Supplement: Supplementary file 1 — Table S1: List of the functional categories used for classification of eukaryotic and prokaryotic DEGs in the manually curated database of L. capensis . Table S2: Relative frequency of functional gene categories (as listed in Table S1) among differentially expressed eukaryotic genes (DEGs) identified in the gill tissues of L. capensis exposed to varying oxygen conditions. Table S3: Relative frequency of functional gene categories (as listed in Table S1) among differentially expressed prokaryotic genes (DEGs) identified in the gill tissues of L. capensis exposed to varying oxygen conditions. Table S5: Results of the Reactome analysis of the gill transcriptome of L. capensis , displaying only significantly up‐ or downregulated pathways (FDR < 0.1). No significantly enriched pathways were identified in the following comparisons: R24 (24 h of reoxygenation) versus normoxia (N), R1 (1 h of reoxygenation) versus hypoxia (H), and R24 versus hypoxia. Additionally, no significantly downregulated pathways were found in the R1 versus N comparison. “# Genes found” refers to the number of identified DEGs, while “# Genes total” refers to the total number of genes in the respective pathway. Rxn—reactions. For the sake of completeness, we report all pathways identified as significantly enriched by Reactome analysis. Pathways that are specific to vertebrates and are unlikely to be relevant in molluscs are indicated in italics. Table S6: Results of the Reactome analysis of the digestive gland transcriptome of L. capensis , displaying only significantly up‐ or downregulated pathways (FDR < 0.1). No significantly downregulated pathways were identified in the following comparisons: hypoxia (H) versus normoxia (N), R24 (24 h of reoxygenation) versus N. No significantly downregulated pathways were identified in the following comparisons: R24 versus N and R24 versus A. “# Genes found” refers to the number of identified DEGs, while “# Genes total” refers to the total number of genes in the [file MEC-34-e70194-s001.docx]

**Supplementary materials for manuscript**

**Unraveling Hypoxia Tolerance: Transcriptomic and Metabolic Insights from *Lucinoma capensis* in an Oxygen Minimum Zone**

Inna M. Sokolova^1,2*^, Eugene P. Sokolov^1^, Helen Piontkivska^3,4^, Stefan Timm^5^, Katherine Amorim^6^, Michael L. Zettler^6*^

^1^ Department of Marine Biology, Institute for Biological Sciences, University of Rostock, Rostock, Germany

^2^ Department of Maritime Systems, Interdisciplinary Faculty, University of Rostock, Rostock, Germany

^3^ Department of Biological Sciences, Kent State University, Kent, OH, USA

^4^ Brain Health Research Institute, Kent State University, Kent, OH, USA

^5^ Department of Plant Physiology, Institute for Biological Sciences, University of Rostock, Rostock, Germany

^6^ Department of Biological Oceanography, Leibniz Institute for Baltic Sea Research Warnemünde, Rostock, Germany

**Supplementary Text 1**

**Sequence Assembly and Transcriptome Analyses**

The RNA extraction, polyA enrichment, mRNA library preparation and next-generation sequencing on Illumina NovaSeq 6000 PE150 platform were carried out by Novogene GmbH (Munich, Germany). After adapter trimming, 2 to 4 % of low-quality reads (such as those with over 10% of uncertain nucleotides, or when base quality of less than 5 comprises more that 50 percent of the read) were filtered out, with an average 3% filtered out reads per sample (see Zenodo 10.5281/zenodo.16367847 for the number of reads and other assembly details). The raw sequencing reads were then processed and assembled into transcripts using Trinity 2.6.6 (https://github.com/trinityrnaseq/trinityrnaseq/) (Grabherr et al., 2011). Gene-level counts were computed with CORSET 4.6 (Davidson and Oshlack, 2014) that performs hierarchical clustering of contigs by taking into account shared reads and expression patterns, and selecting the longest transcripts of each cluster as unigenes. (https://github.com/Oshlack/Corset/wiki). BUSCO 3.0.2 was run in transcriptome mode using the Metazoa odb10 lineage dataset, with default parameters. Only the longest isoform per gene was included in the analysis. Supplementary data for transcriptome assembly parameters, including putative functional annotations, Trinity assembly statistics, including N50 and N90, and lengths of transcript and unigene are available in Zenodo 10.5281/zenodo.16367847. We would like to point out the inherent limitations of the reported transcriptome assembly, as indicated by a high percentage of missing BUSCOs, where the de novo assembly represents only the expressed genes from the two tissues samples of an adult stage under specific conditions, and thus, can be expected to be missing genes that are not expressed in these tissues and/or expressed under different conditions or life stages. Moreover, our target species is phylogenetically distant from the reference set, with at least ~494 million years of divergence time between *Lucinoma* and other bivalves with complete and annotated genomes (Bieler et al., 2014; Kumar et al., 2022), and thus, its genome can be expected to also harbor some divergence from the set of conserved BUSCO orthologs, whether at the sequence level or at the level of missing genes. Nonetheless, despite the relatively low BUSCO completeness, other metrics indicate that the assembled transcriptome is of sufficient quality for downstream analyses, including recovering 68,267 transcripts in the gills and 55,981 transcripts in the digestive gland, with the N50 and N90 of 748 and 363 base pairs, respectively.

Gene expression levels were estimated with RSEM (Li and Dewey, 2011) based on transcript abundance using the Corset-filtered Trinity-reconstructed transcriptome as a reference. Read counts were converted into FPKM (Fragments Per Kilobase of transcript sequence per Million base pairs sequenced), an approach that adjusts for both sequencing depth and gene length in fragment counts. Prediction of protein-coding sequences was performed using BLAST (Altschul et al., 1997) against NCBI NR and SwissProt databases (Genbank and UniProt Releases 239 and 2020_04, respectively) for CDS extraction with cut-off e values of 1e-5, and ESTScan with default values (Nagaraj et al., 2007) for unigenes without hits in BLAST.

Differential gene expression analysis was performed with DESeq2 (Love et al., 2014), using read counts as input data. Benjamini–Hochberg correction (Benjamini and Hochberg, 1995) was used to adjust for multiple tests, with the cut-off p-adjusted value of 0.05 and |log2FoldChange| > 0.58 used to identify differentially expressed genes (DEGs) that showed at least 50% change in expression level in either direction.

Gene functional enrichment analysis was performed to identify biological functions significantly associated with DEGs using Gene Ontology (GO) as implemented in GOseq (https://bioconductor.org/packages/release/bioc/html/goseq.html), with corrected p values < 0.05. For a more in-depth insight into the functional transcriptomic response to hypoxia-reoxygenation, we manually curated the list of DEGs by assigning all annotated DEGs to 64 functional categories for eukaryotic transcripts and 41 categories for prokaryotic transcripts (Supplementary Table 1). The transcripts annotated as genes with unknown functions were omitted. Each DEG was manually reviewed and assigned to a functional category based on its annotated function, supported by homology searches against the NCBI nucleotide database and the SwissProt protein database (see Zenodo 10.5281/zenodo.16367847 for details of manual annotation). The likely origin of each gene—prokaryotic or eukaryotic—was determined through sequence homology comparisons with relevant reference databases. To ensure non-redundancy, genes sharing identical NCBI or SwissProt accession numbers were flagged, reviewed, and, where appropriate, merged or removed. This process yielded a final non-redundant list of DEGs. Functional categories were then quantified by counting the number of genes assigned to each category.

***Reactome analysis.*** Since Reactome overrepresentation algorithm makes pathway predictions based on the human genomic database (Milacic et al., 2023), we converted non-human gene IDs to the Ensembl IDs of respective human homologs using BLASTP search against protein sequences of GRCh38 human genome assembly (https://ftp.ensembl.org/pub/release-110/fasta/homo_sapiens/pep/). We used relatively relaxed cut-off e value of 1e-5 to capture even distant homologs. Only eukaryotic transcripts of *L. capensis* were included in search of homologs.

Once we identified respective human homologs, we applied two cut-off thresholds for Reactome pathways overrepresentation interpretation: a more conservative FDR < 0.05 and a more relaxed FDR < 0.1. Setting the FDR at 0.1, instead of the traditional 0.05, increases sensitivity, allowing us to capture more potentially relevant metabolic pathways. In exploratory studies or large-scale analyses with numerous tests, such as ours, a less stringent FDR helps detect subtle but meaningful patterns, minimizing the risk of overlooking true positives (Benjamini and Hochberg, 1995). This approach is particularly valuable when the focus is on identifying promising leads for further investigation, rather than confirming established hypotheses. Reactome analyses were performed using version 90 (released September 2024).

We would like to acknowledge that the use of Reactome, which is primarily focused on human data for pathway annotations, has certain limitations when applied to non-model organisms. One of the main limitations is its bias toward human disease-related pathways, which may not be directly relevant to non-human species. Some highly conserved molluscan gene homologs may appear associated with these human disease pathways, potentially leading to misinterpretation. To avoid ambiguity, we have manually curated the Reactome results to focus on pathways that have broad physiological relevance and are evolutionarily conserved across animals (see Supplementary Tables 5 and 6).

References:

Altschul SF, Madden TL, Schäffer AA, Zhang J, Zhang Z, Miller W, et al. Gapped BLAST and PSI-BLAST: a new generation of protein database search programs. *Nucleic Acids Research* 1997; 25: 3389-3402.

Benjamini Y, Hochberg Y. Controlling the False Discovery Rate: A Practical and Powerful Approach to Multiple Testing. *Journal of the Royal Statistical Society: Series B (Methodological)* 1995; 57: 289-300.

Bieler, R., Mikkelsen, P.M., Collins, T.M., Glover, E.A., González, V.L., Graf, D.L., Harper, E.M., Healy, J., Kawauchi, G.Y., Sharma, P.P. and Staubach, S., 2014. Investigating the Bivalve Tree of Life–an exemplar-based approach combining molecular and novel morphological characters. *Invertebrate Systematics*, *28*(1), pp.32-115.

Davidson NM, Oshlack A. Corset: enabling differential gene expression analysis for de novoassembled transcriptomes. *Genome Biology* 2014; 15: 410.

Grabherr MG, Haas BJ, Yassour M, Levin JZ, Thompson DA, Amit I, et al. Full-length transcriptome assembly from RNA-Seq data without a reference genome. *Nat Biotechnol* 2011; 29: 644-52.

Kumar, S., Suleski, M., Craig, J.M., Kasprowicz, A.E., Sanderford, M., Li, M., Stecher, G. and Hedges, S.B., 2022. TimeTree 5: an expanded resource for species divergence times. *Molecular biology and evolution*, *39*(8), p.msac174.

Li B, Dewey CN. RSEM: accurate transcript quantification from RNA-Seq data with or without a reference genome. *BMC Bioinformatics* 2011; 12: 323.

Love MI, Huber W, Anders S. Moderated estimation of fold change and dispersion for RNA-seq data with DESeq2. *Genome Biology* 2014; 15: 550.

Milacic M, Beavers D, Conley P, Gong C, Gillespie M, Griss J, et al. The Reactome Pathway Knowledgebase 2024. *Nucleic Acids Research* 2023; 52: D672-D678.

**Supplementary Text 2**

**Metabolite Analyses**

Metabolite concentrations in clam tissues were measured using high-performance liquid chromatography-mass spectrometry as described earlier (Bruhns et al., 2023). Briefly, metabolites were extracted from the gill (mean tissue mass: 37.0±3.5 mg, range 3.5-98.9 mg) and the digestive gland (mean tissue mass: 35.4±5.4 mg, range 2-95.5 mg) tissue, homogenized in 1 ml of ice-cold 80% ethanol containing 1 μg ml^−1^ of 2-(N-morpholino)ethanesulfonic acid (MES) as an internal standard. Each sample was derived from individual clams. The homogenates were centrifuged at 13,000 ×g for 10 min at 4°C to remove debris. The resulting supernatants were freeze-dried using a cold trap (Unicryo MC 2 L, UniEquip, Germany) and stored at −80°C for future analysis. Prior to measurement, extracts were reconstituted in LC-MS-grade water (ROTISOLV, Carl Roth) and passed through sterile 0.2 μm filters (Omnifix-F, Braun, Germany). Metabolite measurements were conducted on a Shimadzu LCMS-8050 high-performance liquid chromatography-mass spectrometer. Metabolites were identified and quantified using the built-in LC-MS/MS software package for primary metabolites (Version 2, Shimadzu, P/N 225–24,862-92) and LabSolutions software (Shimadzu). Calibration was performed using metabolite-specific standards from Merck, with normalization based on the internal standard (2-(N-morpholino)ethanesulfonic acid).

**Supplementary Table 1.** List of the functional categories used for classification of eukaryotic and prokaryotic DEGs in the manually curated database of *L. capensis*.

| Eukaryotic DEGs | Prokaryotic DEGs |
| --- | --- |
| Acid-base regulation | Amino acid metabolism |
| Adhesion | Antibiotic metabolism |
| Amino acid metabolism | Apoptosis |
| Angiopoesis | ATP synthase |
| Apoptosis | Carbohydrate metabolism |
| ATP synthase (incl. assembly) | Cell division |
| Autophagy | Cell motility |
| Blood clotting | Cell wall |
| Ca metabolism | Chaperones |
| Carbohydrate metabolism | CO_2_ fixation |
| Cell division & differentiation | Cofactor biosynthesis & metabolism |
| Chaperones | Deacetylase |
| Ciliary activity | Detoxification |
| Cofactor metabolism & biosynthesis | DNA maintenance |
| Cytoskeleton | ETS complexes (incl. assembly) |
| Detoxification | Glycolysis |
| Development | Hydrocarbon & alcohol metabolism |
| Digestion | Ion transport |
| DNA maintenance | Lipid metabolism |
| Endocrine function | Lipopolysaccharide biosynthesis |
| Endocytosis | Membrane transport |
| ETS complexes (incl. assembly) | Metal homeostasis |
| Extracellular matrix | Mobile genetic elements |
| Glycoprotein metabolism | Nitrogen fixation |
| Glycosphingolipid biosynthesis | NO metabolism |
| GSH metabolism | Nucleotide metabolism |
| Histone modification | Oxidoreductases |
| Immunity | Peptidoglycan synthesis |
| Inflammation | Phosphate metabolism and transport |
| Ion transport | Protein synthesis |
| Iron homeostasis | Protein transport |
| Ketone metabolism | Proteolysis & protein repair |
| Lipid metabolism | Recombination |
| Locomotion | Redox homeostasis |
| Membrane and substrate transport | RNA degradation |
| Membrane function | Signaling |
| miRNA transport | Sulfur metabolism |
| Mobile genetic elements | Transcription regulation |
| Neural function | Tricarboxylic acid cycle (TCA) |
| NO metabolism | Urea metabolism |
| Nuclear transport | Virulence factor |
| Nucleotide metabolism |  |
| Oogenesis |  |
| Organelle biogenesis |  |
| Osmoregulation |  |
| Oxidoreductases |  |
| Phosphate metabolism and transport |  |
| Post-translational protein modifications |  |
| Protein synthesis |  |
| Protein transport |  |
| Proteolysis | |
| Redox homeostasis |  |
| RNA metabolism |  |
| Secretion |  |
| Signaling |  |
| Spermatogenesis |  |
| Sphingolipid metabolism |  |
| Steroid metabolism & biosynthesis |  |
| Stress response |  |
| Sulfur metabolism |  |
| Transcription regulation | |
| Tricarboxylic acid cycle (TCA) |  |
| Urea metabolism |  |
| Vision |  |

**Supplementary Table 2.** Relative frequency of functional gene categories (as listed in Supplementary Table 1) among differentially expressed eukaryotic genes (DEGs) identified in the gill tissues of *L. capensis* exposed to varying oxygen conditions.

The table displays the percentage of total DEGs for each category and the cumulative percentage (Cum. %) of categories, ranked in descending order of DEG count.

| **A. Downregulated in hypoxia vs. normoxia (819 DEGs)** | | | | **B. Upregulated in hypoxia vs. normoxia (236 DEGs)** | | | |
| --- | --- | --- | --- | --- | --- | --- | --- |
| **Functional group** | **# DEGs** | **% of total** | **Cum.%** | **Functional group** | **# DEGs** | **% of total** | **Cum.%** |
| Protein synthesis | 150 | 18.3 | 18.3 | Proteolysis | 25 | 10.6 | 10.6 |
| Proteolysis | 62 | 7.6 | 25.9 | Transcription regulation | 18 | 7.6 | 18.2 |
| Mobile genetic elements (transposition) | 58 | 7.1 | 33.0 | Immunity | 17 | 7.2 | 25.4 |
| Immunity | 52 | 6.3 | 39.3 | Cytoskeleton | 17 | 7.2 | 32.6 |
| Signaling | 47 | 5.7 | 45.1 | Carbohydrate metabolism | 15 | 6.4 | 39.0 |
| Transcription regulation | 40 | 4.9 | 49.9 | DNA maintenance | 13 | 5.5 | 44.5 |
| ETS complexes (incl. assembly) | 32 | 3.9 | 53.8 | ECM | 13 | 5.5 | 50.0 |
| Detoxification | 23 | 2.8 | 56.7 | Protein synthesis | 12 | 5.1 | 55.1 |
| Neural function | 23 | 2.8 | 59.5 | Chaperone | 10 | 4.2 | 59.3 |
| Carbohydrate metabolism | 22 | 2.7 | 62.1 | Signaling | 8 | 3.4 | 62.7 |
| DNA maintenance | 21 | 2.6 | 64.7 | Adhesion | 7 | 3.0 | 65.7 |
| AA metabolism | 21 | 2.6 | 67.3 | Mobile genetic elements (transposition) | 7 | 3.0 | 68.6 |
| Lipid metabolism | 19 | 2.3 | 69.6 | Redox homeostasis | 7 | 3.0 | 71.6 |
| RNA metabolism | 16 | 2.0 | 71.6 | ETS complexes (incl. assembly) | 6 | 2.5 | 74.2 |
| Cytoskeleton | 15 | 1.8 | 73.4 | Cell division & differentiation | 5 | 2.1 | 76.3 |
| ECM | 14 | 1.7 | 75.1 | Nucleotide metabolism | 5 | 2.1 | 78.4 |
| Cell division & differentiation | 13 | 1.6 | 76.7 | Endocytosis | 4 | 1.7 | 80.1 |
| Redox homeostasis | 13 | 1.6 | 78.3 | Locomotion | 4 | 1.7 | 81.8 |
| Chaperone | 12 | 1.5 | 79.7 | Membrane and substrate transport | 4 | 1.7 | 83.5 |
| Apoptosis | 12 | 1.5 | 81.2 | AA metabolism | 3 | 1.3 | 84.7 |
| Membrane and substrate transport | 12 | 1.5 | 82.7 | Ca metabolism | 3 | 1.3 | 86.0 |
| Cofactor metabolism & biosynthesis | 10 | 1.2 | 83.9 | Detoxification | 3 | 1.3 | 87.3 |
| Adhesion | 9 | 1.1 | 85.0 | Inflammation | 3 | 1.3 | 88.6 |
| Ca metabolism | 9 | 1.1 | 86.1 | Spermatogenesis | 3 | 1.3 | 89.8 |
| Nucleotide metabolism | 9 | 1.1 | 87.2 | Neural function | 3 | 1.3 | 91.1 |
| Locomotion | 8 | 1.0 | 88.2 | RNA metabolism | 3 | 1.3 | 92.4 |
| Histone modification | 7 | 0.9 | 89.0 | Development | 2 | 0.8 | 93.2 |
| Sulfur metabolism | 7 | 0.9 | 89.9 | Ciliary activity | 2 | 0.8 | 94.1 |
| Protein transport | 6 | 0.7 | 90.6 | Ion transport | 2 | 0.8 | 94.9 |
| Ciliary activity | 5 | 0.6 | 91.2 | Cofactor metabolism & biosynthesis | 2 | 0.8 | 95.8 |
| Membrane function | 5 | 0.6 | 91.8 | Sulfur metabolism | 2 | 0.8 | 96.6 |
| Spermatogenesis | 5 | 0.6 | 92.4 | Apoptosis | 1 | 0.4 | 97.0 |
| Sphingolipid metabolism | 5 | 0.6 | 93.0 | Glycoprotein metabolism | 1 | 0.4 | 97.5 |
| ATP synthase (incl. assembly) | 5 | 0.6 | 93.7 | Glycosphingolipid biosynthesis | 1 | 0.4 | 97.9 |
| Inflammation | 4 | 0.5 | 94.1 | Lipid metabolism | 1 | 0.4 | 98.3 |
| Ion transport | 4 | 0.5 | 94.6 | Membrane function | 1 | 0.4 | 98.7 |
| PTM | 4 | 0.5 | 95.1 | Nuclear transport | 1 | 0.4 | 99.2 |
| Stress response | 4 | 0.5 | 95.6 | Oogenesis | 1 | 0.4 | 99.6 |
| Steroid metabolism & biosynthesis | 4 | 0.5 | 96.1 | Oxidoreductases | 1 | 0.4 | 100.0 |
| Phosphate metabolism and transport | 3 | 0.4 | 96.5 |  |  |  |  |
| Acid-base regulation | 3 | 0.4 | 96.8 |  |  |  |  |
| Nuclear transport | 3 | 0.4 | 97.2 |  |  |  |  |
| Blood clotting | 2 | 0.2 | 97.4 |  |  |  |  |
| Development | 2 | 0.2 | 97.7 |  |  |  |  |
| Endocytosis | 2 | 0.2 | 97.9 |  |  |  |  |
| Angiopoesis | 2 | 0.2 | 98.2 |  |  |  |  |
| Endocrine function | 2 | 0.2 | 98.4 |  |  |  |  |
| Urea metabolism | 2 | 0.2 | 98.7 |  |  |  |  |
| Iron homeostasis | 2 | 0.2 | 98.9 |  |  |  |  |
| NO metabolism | 1 | 0.1 | 99.0 |  |  |  |  |
| Autophagy | 1 | 0.1 | 99.1 |  |  |  |  |
| Digestion | 1 | 0.1 | 99.3 |  |  |  |  |
| Glycoprotein metabolism | 1 | 0.1 | 99.4 |  |  |  |  |
| Ketone metabolism | 1 | 0.1 | 99.5 |  |  |  |  |
| miRNA transport | 1 | 0.1 | 99.6 |  |  |  |  |
| Vision? | 1 | 0.1 | 99.8 |  |  |  |  |
| Organelle biogenesis | 1 | 0.1 | 99.9 |  |  |  |  |
| TCA | 1 | 0.1 | 100.0 |  |  |  |  |
| **C. Downregulated in 1 h recovery vs. normoxia (409 DEGs)** | | | | **D. Upregulated in 1 h recovery vs. normoxia (289 DEGs)** | | | |
| **Functional group** | **# DEGs** | **% of total** | **Cum. %** | **Functional group** | **# DEGs** | **% of total** | **Cum.%** |
| Proteolysis incl. ubiquitin-proteasome pathway | 49 | 12.0 | 12.0 | Proteolysis incl. ubiquitin-proteasome pathway | 27 | 9.3 | 9.3 |
| Mobile genetic elements (transposition) | 43 | 10.5 | 22.5 | ECM | 19 | 6.6 | 15.9 |
| Immunity | 30 | 7.3 | 29.8 | Mobile genetic elements (transposition) | 17 | 5.9 | 21.8 |
| Transcription regulation & transcription factors | 25 | 6.1 | 35.9 | Immunity | 17 | 5.9 | 27.7 |
| Protein synthesis | 23 | 5.6 | 41.6 | Transcription regulation & transcription factors | 17 | 5.9 | 33.6 |
| Signaling | 19 | 4.6 | 46.2 | Signaling | 16 | 5.5 | 39.1 |
| Apoptosis | 16 | 3.9 | 50.1 | Cytoskeleton | 14 | 4.8 | 43.9 |
| DNA maintenance | 15 | 3.7 | 53.8 | Protein synthesis | 12 | 4.2 | 48.1 |
| Detoxification | 12 | 2.9 | 56.7 | Lipid metabolism | 11 | 3.8 | 51.9 |
| Lipid metabolism | 12 | 2.9 | 59.7 | DNA maintenance | 10 | 3.5 | 55.4 |
| Cytoskeleton | 10 | 2.4 | 62.1 | Adhesion | 9 | 3.1 | 58.5 |
| Neural function | 10 | 2.4 | 64.5 | Chaperone | 8 | 2.8 | 61.2 |
| Membrane and substrate transport | 10 | 2.4 | 67.0 | Neural function | 9 | 3.1 | 64.4 |
| Cell division & differentiation | 9 | 2.2 | 69.2 | Carbohydrate metabolism | 7 | 2.4 | 66.8 |
| Carbohydrate metabolism | 9 | 2.2 | 71.4 | Cell division & differentiation | 7 | 2.4 | 69.2 |
| Nucleotide metabolism | 8 | 2.0 | 73.3 | RNA metabolism | 7 | 2.4 | 71.6 |
| Redox homeostasis | 8 | 2.0 | 75.3 | AA metabolism | 6 | 2.1 | 73.7 |
| AA metabolism | 7 | 1.7 | 77.0 | Locomotion | 6 | 2.1 | 75.8 |
| RNA metabolism | 7 | 1.7 | 78.7 | Detoxification | 5 | 1.7 | 77.5 |
| Chaperone | 6 | 1.5 | 80.2 | Nucleotide metabolism | 5 | 1.7 | 79.2 |
| ECM | 6 | 1.5 | 81.7 | Redox homeostasis | 4 | 1.4 | 80.6 |
| Histone modification | 6 | 1.5 | 83.1 | Inflammation | 4 | 1.4 | 82.0 |
| Adhesion | 5 | 1.2 | 84.4 | Development | 4 | 1.4 | 83.4 |
| Oxidoreductases | 5 | 1.2 | 85.6 | Membrane and substrate transport | 4 | 1.4 | 84.8 |
| Inflammation | 4 | 1.0 | 86.6 | Ca metabolism | 3 | 1.0 | 85.8 |
| Locomotion | 4 | 1.0 | 87.5 | Spermatogenesis | 3 | 1.0 | 86.9 |
| Cofactor metabolism & biosynthesis | 4 | 1.0 | 88.5 | Nuclear transport | 3 | 1.0 | 87.9 |
| ETS complexes (incl. assembly) | 4 | 1.0 | 89.5 | Ciliary activity | 3 | 1.0 | 88.9 |
| Ca metabolism | 3 | 0.7 | 90.2 | Cofactor metabolism & biosynthesis | 3 | 1.0 | 90.0 |
| Ion transport | 3 | 0.7 | 91.0 | ETS complexes (incl. assembly) | 3 | 1.0 | 91.0 |
| Membrane function | 3 | 0.7 | 91.7 | Endocytosis | 2 | 0.7 | 91.7 |
| Nuclear transport | 3 | 0.7 | 92.4 | Glycoprotein metabolism | 2 | 0.7 | 92.4 |
| Sulfur metabolism | 3 | 0.7 | 93.2 | Histone modification | 2 | 0.7 | 93.1 |
| Endocytosis | 3 | 0.7 | 93.9 | Phosphate metabolism and transport | 2 | 0.7 | 93.8 |
| Steroid metabolism & biosynthesis | 3 | 0.7 | 94.6 | Protein transport | 2 | 0.7 | 94.5 |
| Iron homeostasis | 3 | 0.7 | 95.4 | Stress response | 2 | 0.7 | 95.2 |
| Acid-base regulation | 2 | 0.5 | 95.8 | Sulfur metabolism | 2 | 0.7 | 95.8 |
| Protein transport | 2 | 0.5 | 96.3 | Osmoregulation | 2 | 0.7 | 96.5 |
| Stress response | 2 | 0.5 | 96.8 | Apoptosis | 2 | 0.7 | 97.2 |
| Autophagy | 1 | 0.2 | 97.1 | Oxidoreductases | 2 | 0.7 | 97.9 |
| Ciliary activity | 1 | 0.2 | 97.3 | Autophagy | 1 | 0.3 | 98.3 |
| Glycoprotein metabolism | 1 | 0.2 | 97.6 | Ion transport | 1 | 0.3 | 98.6 |
| Ketone metabolism | 1 | 0.2 | 97.8 | Iron homeostasis | 1 | 0.3 | 99.0 |
| Oogenesis | 1 | 0.2 | 98.0 | PTM | 1 | 0.3 | 99.3 |
| Organelle biogenesis | 1 | 0.2 | 98.3 | Sphingolipid metabolism | 1 | 0.3 | 99.7 |
| Osmoregulation | 1 | 0.2 | 98.5 | Steroid metabolism & biosynthesis | 1 | 0.3 | 100.0 |
| Phosphate metabolism and transport | 1 | 0.2 | 98.8 |  |  |  |  |
| Spermatogenesis | 1 | 0.2 | 99.0 |  |  |  |  |
| Sphingolipid metabolism | 1 | 0.2 | 99.3 |  |  |  |  |
| Urea metabolism | 1 | 0.2 | 99.5 |  |  |  |  |
| ATP synthase (incl. assembly) | 1 | 0.2 | 99.8 |  |  |  |  |
| TCA | 1 | 0.2 | 100.0 |  |  |  |  |
| **E. Downregulated in 24 h recovery vs. normoxia (261 DEGs)** | | | | **F. Upregulated in 24 h recovery vs. normoxia (328 DEGs)** | | | |
| **Functional group** | **# DEGs** | **% of total** | **Cum. %** | **Functional group** | **# DEGs** | **% of total** | **Cum. %** |
| Immunity | 29 | 11.1 | 11.1 | Proteolysis incl. ubiquitin-proteasome pathway | 27 | 8.2 | 8.2 |
| Proteolysis incl. ubiquitin-proteasome pathway | 23 | 8.8 | 19.9 | Transcription regulation & transcription factors | 25 | 7.6 | 15.9 |
| Protein synthesis | 20 | 7.7 | 27.6 | DNA maintenance | 23 | 7.0 | 22.9 |
| Mobile genetic elements (transposition) | 18 | 6.9 | 34.5 | Mobile genetic elements (transposition) | 20 | 6.1 | 29.0 |
| Chaperone | 11 | 4.2 | 38.7 | Signaling | 17 | 5.2 | 34.1 |
| Transcription regulation & transcription factors | 11 | 4.2 | 42.9 | Locomotion | 14 | 4.3 | 38.4 |
| Neural function | 10 | 3.8 | 46.7 | Cytoskeleton | 12 | 3.7 | 42.1 |
| Lipid metabolism | 8 | 3.1 | 49.8 | Protein synthesis | 12 | 3.7 | 45.7 |
| ECM | 7 | 2.7 | 52.5 | RNA metabolism | 12 | 3.7 | 49.4 |
| Detoxification | 7 | 2.7 | 55.2 | Immunity | 11 | 3.4 | 52.7 |
| Carbohydrate metabolism | 7 | 2.7 | 57.9 | Adhesion | 10 | 3.0 | 55.8 |
| Signaling | 6 | 2.3 | 60.2 | Chaperone | 9 | 2.7 | 58.5 |
| AA metabolism | 6 | 2.3 | 62.5 | ECM | 9 | 2.7 | 61.3 |
| Cell division & differentiation | 6 | 2.3 | 64.8 | Cell division & differentiation | 9 | 2.7 | 64.0 |
| Locomotion | 6 | 2.3 | 67.0 | Ciliary activity | 9 | 2.7 | 66.8 |
| DNA maintenance | 6 | 2.3 | 69.3 | Neural function | 9 | 2.7 | 69.5 |
| RNA metabolism | 6 | 2.3 | 71.6 | PTM | 8 | 2.4 | 72.0 |
| Histone modification | 5 | 1.9 | 73.6 | Histone modification | 7 | 2.1 | 74.1 |
| Ion transport | 5 | 1.9 | 75.5 | Spermatogenesis | 7 | 2.1 | 76.2 |
| Endocytosis | 5 | 1.9 | 77.4 | Carbohydrate metabolism | 7 | 2.1 | 78.4 |
| Apoptosis | 4 | 1.5 | 78.9 | Stress response | 6 | 1.8 | 80.2 |
| Cytoskeleton | 4 | 1.5 | 80.5 | Ca metabolism | 6 | 1.8 | 82.0 |
| GSH metabolism | 3 | 1.1 | 81.6 | Endocytosis | 5 | 1.5 | 83.5 |
| Oogenesis | 3 | 1.1 | 82.8 | ETS complexes (incl. assembly) | 5 | 1.5 | 85.1 |
| PTM | 3 | 1.1 | 83.9 | Development | 4 | 1.2 | 86.3 |
| Iron homeostasis | 3 | 1.1 | 85.1 | Lipid metabolism | 4 | 1.2 | 87.5 |
| Ciliary activity | 3 | 1.1 | 86.2 | Nucleotide metabolism | 4 | 1.2 | 88.7 |
| ETS complexes (incl. assembly) | 3 | 1.1 | 87.4 | Apoptosis | 3 | 0.9 | 89.6 |
| Acid-base regulation | 2 | 0.8 | 88.1 | Detoxification | 3 | 0.9 | 90.5 |
| Autophagy | 2 | 0.8 | 88.9 | Ion transport | 3 | 0.9 | 91.5 |
| Development | 2 | 0.8 | 89.7 | Membrane and substrate transport | 3 | 0.9 | 92.4 |
| Ketone metabolism | 2 | 0.8 | 90.4 | Nuclear transport | 3 | 0.9 | 93.3 |
| Oxidoreductases | 2 | 0.8 | 91.2 | Redox homeostasis | 3 | 0.9 | 94.2 |
| Protein transport | 2 | 0.8 | 92.0 | Membrane function | 2 | 0.6 | 94.8 |
| PTM | 2 | 0.8 | 92.7 | Organelle biogenesis | 2 | 0.6 | 95.4 |
| Spermatogenesis | 2 | 0.8 | 93.5 | Secretion | 2 | 0.6 | 96.0 |
| Membrane and substrate transport | 2 | 0.8 | 94.3 | Membrane and substrate transport | 2 | 0.6 | 96.6 |
| Sulfur metabolism | 2 | 0.8 | 95.0 | ATP synthase (incl. assembly) | 2 | 0.6 | 97.3 |
| Nucleotide metabolism | 2 | 0.8 | 95.8 | AA metabolism | 2 | 0.6 | 97.9 |
| Adhesion | 1 | 0.4 | 96.2 | Cofactor metabolism & biosynthesis | 2 | 0.6 | 98.5 |
| Ca metabolism | 1 | 0.4 | 96.6 | Oxidoreductases | 2 | 0.6 | 99.1 |
| Cofactor metabolism & biosynthesis | 1 | 0.4 | 96.9 | Glycoprotein metabolism | 1 | 0.3 | 99.4 |
| Endocrine function | 1 | 0.4 | 97.3 | Inflammation | 1 | 0.3 | 99.7 |
| Nuclear transport | 1 | 0.4 | 97.7 | Protein transport | 1 | 0.3 | 100.0 |
| Phosphate metabolism and transport | 1 | 0.4 | 98.1 |  |  |  |  |
| Secretion | 1 | 0.4 | 98.5 |  |  |  |  |
| Sphingolipid metabolism | 1 | 0.4 | 98.9 |  |  |  |  |
| Steroid metabolism & biosynthesis | 1 | 0.4 | 99.2 |  |  |  |  |
| Stress response | 1 | 0.4 | 99.6 |  |  |  |  |
| Urea metabolism | 1 | 0.4 | 100.0 |  |  |  |  |

**Supplementary Table 3.** Relative frequency of functional gene categories (as listed in Supplementary Table 1) among differentially expressed prokaryotic genes (DEGs) identified in the gill tissues of *L. capensis* exposed to varying oxygen conditions.

The table displays the percentage of total DEGs for each category and the cumulative percentage (Cum. %) of categories, ranked in descending order of DEG count.

| 1. **Downregulated in hypoxia vs. normoxia (40)** | | | | 1. **Upregulated in hypoxia vs. normoxia (139)** | | | |
| --- | --- | --- | --- | --- | --- | --- | --- |
| **Functional group** | **# DEGs** | **% of total** | **Cum.%** | **Functional group** | **# DEGs** | **% of total** | **Cum.%** |
| Antibiotic metabolism | 4 | 10 | 10 | Sulfur metabolism | 28 | 20.1 | 20.1 |
| Amino acid metabolism | 4 | 10 | 20 | CO2 fixation | 18 | 12.9 | 33.1 |
| Proteolysis & protein repair | 3 | 7.5 | 27.5 | ETS | 16 | 11.5 | 44.6 |
| Chaperone | 2 | 5 | 32.5 | Protein synthesis and transport | 15 | 10.8 | 55.4 |
| CO2 fixation | 2 | 5 | 37.5 | Amino acid metabolism | 14 | 10.1 | 65.5 |
| Phosphate metabolism | 2 | 5 | 42.5 | Nucleotide metabolism | 6 | 4.3 | 69.8 |
| Mobile genetic elements | 2 | 5 | 47.5 | NO metabolism | 4 | 2.9 | 72.7 |
| Hydrocarbon & alcohol metabolism | 2 | 5 | 52.5 | Proteolysis & protein repair | 4 | 2.9 | 75.5 |
| Cofactor biosynthesis & metabolism | 2 | 5 | 57.5 | Membrane transport | 4 | 2.9 | 78.4 |
| Transcription regulation | 2 | 5 | 62.5 | ATP synthase | 3 | 2.2 | 80.6 |
| Metal homeostasis | 2 | 5 | 67.5 | Chaperone | 3 | 2.2 | 82.7 |
| Lipopolysaccharide biosynthesis | 2 | 5 | 72.5 | Peptidoglycan synthesis | 3 | 2.2 | 84.9 |
| DNA maintenance | 1 | 2.5 | 75 | Redox homeostasis | 3 | 2.2 | 87.1 |
| ETS | 1 | 2.5 | 77.5 | Cell division | 2 | 1.4 | 88.5 |
| Nitrogen fixation | 1 | 2.5 | 80 | Lipopolysaccharide biosynthesis | 2 | 1.4 | 89.9 |
| NO metabolism | 1 | 2.5 | 82.5 | Phosphate metabolism | 2 | 1.4 | 91.4 |
| Oxidoreductases | 1 | 2.5 | 85 | Signaling | 2 | 1.4 | 92.8 |
| Peptidoglycan synthesis | 1 | 2.5 | 87.5 | Tricarboxylic acid cycle | 2 | 1.4 | 94.2 |
| Protein synthesis and transport | 1 | 2.5 | 90 | DNA maintenance | 2 | 1.4 | 95.7 |
| Recombination | 1 | 2.5 | 92.5 | Transcription regulation | 2 | 1.4 | 97.1 |
| Signaling | 1 | 2.5 | 95 | Lipid metabolism | 1 | 0.7 | 97.8 |
| Tricarboxylic acid cycle | 1 | 2.5 | 97.5 | RNA degradation | 1 | 0.7 | 98.6 |
| Virulence factor | 1 | 2.5 | 100 | Mobile genetic elements (transposition) | 1 | 0.7 | 99.3 |
|  |  |  |  | Virulence factor | 1 | 0.7 | 100.0 |
| 1. **Downregulated in 1 h recovery vs. normoxia (88)** | | | | 1. **Upregulated in 1 h recovery vs. normoxia (52)** | | | |
| **Functional group** | **# DEGs** | **% of total** | **Cum. %** | **Functional group** | **# DEGs** | **% of total** | **Cum. %** |
| Amino acid metabolism | 11 | 12.5 | 12.5 | CO2 fixation | 8 | 15.4 | 15.4 |
| Protein synthesis and transport | 8 | 9.1 | 21.6 | NO metabolism | 6 | 11.5 | 26.9 |
| Mobile genetic elements (transposition) | 5 | 5.7 | 27.3 | Sulfur metabolism | 5 | 9.6 | 36.5 |
| Protein transport | 4 | 4.5 | 31.8 | Transcription regulation | 6 | 11.5 | 48.1 |
| Antibiotic metabolism | 3 | 3.4 | 35.2 | Tricarboxylic acid cycle | 3 | 5.8 | 53.8 |
| Chaperone | 3 | 3.4 | 38.6 | Amino acid metabolism | 3 | 5.8 | 59.6 |
| DNA maintenance | 3 | 3.4 | 42.0 | Antibiotic metabolism | 2 | 3.8 | 63.5 |
| Proteolysis & protein repair | 3 | 3.4 | 45.5 | Membrane transport | 2 | 3.8 | 67.3 |
| Redox homeostasis | 3 | 3.4 | 48.9 | Protein synthesis and transport | 2 | 3.8 | 71.2 |
| Tricarboxylic acid cycle | 3 | 3.4 | 52.3 | Glycolysis | 1 | 1.9 | 73.1 |
| Transcription regulation | 3 | 3.4 | 55.7 | ATP synthase | 1 | 1.9 | 75.0 |
| Cofactor biosynthesis & metabolism | 3 | 3.4 | 59.1 | Chaperone | 1 | 1.9 | 76.9 |
| Carbohydrate metabolism | 2 | 2.3 | 61.4 | Detoxification | 1 | 1.9 | 78.8 |
| Cell wall | 2 | 2.3 | 63.6 | DNA maintenance | 1 | 1.9 | 80.8 |
| CO2 fixation | 2 | 2.3 | 65.9 | ETS | 1 | 1.9 | 82.7 |
| Detoxification | 2 | 2.3 | 68.2 | Ion transport | 1 | 1.9 | 84.6 |
| ETS | 2 | 2.3 | 70.5 | Lipopolysaccharide biosynthesis | 1 | 1.9 | 86.5 |
| Glycolysis | 2 | 2.3 | 72.7 | Nucleotide metabolism | 2 | 3.8 | 90.4 |
| Lipid metabolism | 2 | 2.3 | 75.0 | Oxidoreductases | 1 | 1.9 | 92.3 |
| Membrane transport | 2 | 2.3 | 77.3 | Phosphate metabolism | 1 | 1.9 | 94.2 |
| Metal homeostasis | 2 | 2.3 | 79.5 | Proteolysis & protein repair | 1 | 1.9 | 96.2 |
| Oxidoreductases | 2 | 2.3 | 81.8 | Redox homeostasis | 1 | 1.9 | 98.1 |
| Signaling | 2 | 2.3 | 84.1 | Virulence factor | 1 | 1.9 | 100.0 |
| Nucleotide metabolism | 2 | 2.3 | 86.4 |  |  |  |  |
| Transcription regulation | 2 | 2.3 | 88.6 |  |  |  |  |
| Hydrocarbon and alcohol metabolism | 1 | 1.1 | 89.8 |  |  |  |  |
| Apoptosis | 1 | 1.1 | 90.9 |  |  |  |  |
| Ion transport | 1 | 1.1 | 92.0 |  |  |  |  |
| Lipopolysaccharide biosynthesis | 1 | 1.1 | 93.2 |  |  |  |  |
| NO metabolism | 1 | 1.1 | 94.3 |  |  |  |  |
| Phosphate metabolism and transport | 1 | 1.1 | 95.5 |  |  |  |  |
| Recombination | 1 | 1.1 | 96.6 |  |  |  |  |
| Sulfur metabolism | 1 | 1.1 | 97.7 |  |  |  |  |
| Urea metabolism | 1 | 1.1 | 98.9 |  |  |  |  |
| Virulence factor | 1 | 1.1 | 100.0 |  |  |  |  |
| 1. **Downregulated in 24 h recovery vs. normoxia (18)** | | | | 1. **Upregulated in 24 h recovery vs. normoxia (84)** | | | |
| **Functional group** | **# DEGs** | **% of total** | **Cum. %** | **Functional group** | **# DEGs** | **% of total** | **Cum. %** |
| Transcription regulation | 2 | 11.1 | 11.1 | Protein synthesis and transport | 8 | 9.5 | 9.5 |
| Antibiotic metabolism | 2 | 11.1 | 22.2 | CO2 fixation | 7 | 8.3 | 17.9 |
| Tricarboxylic acid cycle | 2 | 11.1 | 33.3 | Transcription regulation | 7 | 8.3 | 26.2 |
| Virulence factor | 2 | 11.1 | 44.4 | Sulfur metabolism | 7 | 8.3 | 34.5 |
| Cell wall | 2 | 11.1 | 55.6 | ETS | 6 | 7.1 | 41.7 |
| Chaperone | 1 | 5.6 | 61.1 | Chaperone | 5 | 6.0 | 47.6 |
| CO2 fixation | 1 | 5.6 | 66.7 | Amino acid metabolism | 5 | 6.0 | 53.6 |
| DNA maintenance | 1 | 5.6 | 72.2 | Nucleotide metabolism | 4 | 4.8 | 58.3 |
| Lipopolysaccharide biosynthesis | 1 | 5.6 | 77.8 | Cell wall | 4 | 4.8 | 63.1 |
| Phosphate metabolism | 1 | 5.6 | 83.3 | Lipid metabolism | 3 | 3.6 | 66.7 |
| Proteolysis & protein repair | 1 | 5.6 | 88.9 | Oxidoreductases | 3 | 3.6 | 70.2 |
| Sulfur metabolism | 1 | 5.6 | 94.4 | DNA maintenance | 3 | 3.6 | 73.8 |
| Mobile genetic elements (transposition) | 1 | 5.6 | 100.0 | Membrane transport | 3 | 3.6 | 77.4 |
|  |  |  |  | Glycolysis | 2 | 2.4 | 79.8 |
|  |  |  |  | NO metabolism | 2 | 2.4 | 82.1 |
|  |  |  |  | Redox homeostasis | 2 | 2.4 | 84.5 |
|  |  |  |  | Tricarboxylic acid cycle | 2 | 2.4 | 86.9 |
|  |  |  |  | Carbohydrate metabolism | 1 | 1.2 | 88.1 |
|  |  |  |  | Cell division | 1 | 1.2 | 89.3 |
|  |  |  |  | Cell motility | 1 | 1.2 | 90.5 |
|  |  |  |  | Cofactor biosynthesis & metabolism | 1 | 1.2 | 91.7 |
|  |  |  |  | Lipopolysaccharide biosynthesis | 1 | 1.2 | 92.9 |
|  |  |  |  | Phosphate metabolism | 1 | 1.2 | 94.0 |
|  |  |  |  | Proteolysis & protein repair | 1 | 1.2 | 95.2 |
|  |  |  |  | Signaling | 1 | 1.2 | 96.4 |
|  |  |  |  | Stress response | 1 | 1.2 | 97.6 |
|  |  |  |  | Mobile genetic elements (transposition) | 1 | 1.2 | 98.8 |
|  |  |  |  | Virulence factor | 1 | 1.2 | 100.0 |

**Supplementary Table 4.** Relative frequency of functional gene categories (as listed in Supplementary Table 1) among differentially expressed genes (DEGs) identified in the digestive gland tissues of *L. capensis* exposed to varying oxygen conditions.

The table displays the percentage of total DEGs for each category and the cumulative percentage (Cum. %) of categories, ranked in descending order of DEG count.

| 1. **Downregulated in hypoxia vs. normoxia (167)** | | |  | 1. **Upregulated in hypoxia vs. normoxia (382)** | |  |  |
| --- | --- | --- | --- | --- | --- | --- | --- |
| **Functional group** | **# DEGs** | **% of total** | **Cum. %** | **Functional group** | **# DEGs** | **% of total** | Cum.% |
| Protein synthesis | 18 | 10.8 | 10.8 | Immunity | 62 | 16.2 | 16.2 |
| Mobile genetic elements (transposition) | 12 | 7.2 | 18.0 | Proteolysis incl. ubiquitin-proteasome pathway | 38 | 9.9 | 26.2 |
| Proteolysis incl. ubiquitin-proteasome pathway | 11 | 6.6 | 24.6 | ECM | 32 | 8.4 | 34.6 |
| Cell division & differentiation | 8 | 4.8 | 29.3 | Adhesion | 16 | 4.2 | 38.7 |
| Immunity | 7 | 4.2 | 33.5 | Protein synthesis | 16 | 4.2 | 42.9 |
| Signaling | 7 | 4.2 | 37.7 | Neural function | 14 | 3.7 | 46.6 |
| Transcription regulation & transcription factors | 7 | 4.2 | 41.9 | Lipid metabolism | 14 | 3.7 | 50.3 |
| Neural function | 7 | 4.2 | 46.1 | DNA maintenance | 13 | 3.4 | 53.7 |
| Apoptosis | 6 | 3.6 | 49.7 | Cytoskeleton | 11 | 2.9 | 56.5 |
| Ciliary activity | 6 | 3.6 | 53.3 | Signaling | 10 | 2.6 | 59.2 |
| RNA metabolism | 6 | 3.6 | 56.9 | Carbohydrate metabolism | 9 | 2.4 | 61.5 |
| Adhesion | 5 | 3.0 | 59.9 | Mobile genetic elements (transposition) | 9 | 2.4 | 63.9 |
| Cytoskeleton | 5 | 3.0 | 62.9 | Chaperone | 8 | 2.1 | 66.0 |
| ECM | 5 | 3.0 | 65.9 | Cofactor metabolism & biosynthesis | 8 | 2.1 | 68.1 |
| Cofactor metabolism & biosynthesis | 5 | 3.0 | 68.9 | Detoxification | 8 | 2.1 | 70.2 |
| Carbohydrate metabolism | 4 | 2.4 | 71.3 | Locomotion | 8 | 2.1 | 72.3 |
| Ion transport | 4 | 2.4 | 73.7 | Apoptosis | 7 | 1.8 | 74.1 |
| PTM | 4 | 2.4 | 76.0 | Transcription regulation & transcription factors | 7 | 1.8 | 75.9 |
| Membrane and substrate transport | 4 | 2.4 | 78.4 | RNA metabolism | 7 | 1.8 | 77.7 |
| Detoxification | 3 | 1.8 | 80.2 | AA metabolism | 6 | 1.6 | 79.3 |
| Redox homeostasis | 3 | 1.8 | 82.0 | Glycoprotein metabolism | 6 | 1.6 | 80.9 |
| Histone modification | 3 | 1.8 | 83.8 | Ion transport | 6 | 1.6 | 82.5 |
| Lipid metabolism | 3 | 1.8 | 85.6 | Oxidoreductases | 6 | 1.6 | 84.0 |
| Nucleotide metabolism | 3 | 1.8 | 87.4 | Redox homoestasis | 6 | 1.6 | 85.6 |
| Protein transport | 3 | 1.8 | 89.2 | Protein transport | 5 | 1.3 | 86.9 |
| AA metabolism | 3 | 1.8 | 91.0 | Cell division & differentiation | 5 | 1.3 | 88.2 |
| Nuclear transport | 2 | 1.2 | 92.2 | Ca metabolism | 4 | 1.0 | 89.3 |
| Sulfur metabolism | 2 | 1.2 | 93.4 | PTM | 4 | 1.0 | 90.3 |
| Urea metabolism | 2 | 1.2 | 94.6 | ETS complexes (incl. assembly) | 4 | 1.0 | 91.4 |
| DNA maintenance | 1 | 0.6 | 95.2 | Ciliary activity | 3 | 0.8 | 92.1 |
| Chaperone | 1 | 0.6 | 95.8 | Membrane function | 3 | 0.8 | 92.9 |
| Endocytosis | 1 | 0.6 | 96.4 | Stress response | 3 | 0.8 | 93.7 |
| Inflammation | 1 | 0.6 | 97.0 | Inflammation | 2 | 0.5 | 94.2 |
| Locomotion | 1 | 0.6 | 97.6 | Spermatogenesis | 2 | 0.5 | 94.8 |
| Oxidoreductases | 1 | 0.6 | 98.2 | Sphingolipid metabolism | 2 | 0.5 | 95.3 |
| Peptidoglycan synthesis | 1 | 0.6 | 98.8 | Sulfur metabolism | 2 | 0.5 | 95.8 |
| Spermatogenesis | 1 | 0.6 | 99.4 | Osmoregulation | 2 | 0.5 | 96.3 |
| ETS complexes (incl. assembly) | 1 | 0.6 | 100.0 | Angiogenesis | 1 | 0.3 | 96.6 |
|  |  |  |  | Autophagy | 1 | 0.3 | 96.9 |
|  |  |  |  | Endocrine function | 1 | 0.3 | 97.1 |
|  |  |  |  | Secretion | 1 | 0.3 | 97.4 |
|  |  |  |  | Glycoprotein metabolism | 1 | 0.3 | 97.6 |
|  |  |  |  | Histone modification | 1 | 0.3 | 97.9 |
|  |  |  |  | Metal homeostasis | 1 | 0.3 | 98.2 |
|  |  |  |  | Nucleotide metabolism | 1 | 0.3 | 98.4 |
|  |  |  |  | Oogenesis | 1 | 0.3 | 98.7 |
|  |  |  |  | Protein transport | 1 | 0.3 | 99.0 |
|  |  |  |  | Glycoprotein metabolism | 1 | 0.3 | 99.2 |
|  |  |  |  | Steroid metabolism & biosynthesis | 1 | 0.3 | 99.5 |
|  |  |  |  | Membrane and substrate transport | 1 | 0.3 | 99.7 |
|  |  |  |  | Urea metabolism | 1 | 0.3 | 100.0 |
|  |  |  |  |  |  |  |  |
| 1. **Downregulated in 24 h recovery vs. normoxia (144)** | | |  | 1. **Upregulated in 24 h recovery vs. normoxia (384)** | | |  |
| **Functional group** | **# DEGs** | **% of total** | Cum.% | **Functional group** | **# DEGs** | **% of total** | Cum.% |
| Protein synthesis | 18 | 12.5 | 12.5 | Protein synthesis | 39 | 10.2 | 10.2 |
| Immunity | 14 | 9.7 | 22.2 | Immunity | 32 | 8.3 | 18.5 |
| Transposition | 9 | 6.3 | 28.5 | Proteolysis | 25 | 6.5 | 25.0 |
| Adhesion | 7 | 4.9 | 33.3 | Neural function | 20 | 5.2 | 30.2 |
| Proteolysis | 7 | 4.9 | 38.2 | Transcription regulation | 17 | 4.4 | 34.6 |
| Transcription regulation & transcription factors | 7 | 4.9 | 43.1 | Transposition | 17 | 4.4 | 39.1 |
| ECM | 6 | 4.2 | 47.2 | Ciliary function | 16 | 4.2 | 43.2 |
| Cell division & differentiation | 6 | 4.2 | 51.4 | ECM | 15 | 3.9 | 47.1 |
| DNA maintenance | 6 | 4.2 | 55.6 | Signaling | 14 | 3.6 | 50.8 |
| RNA metabolism | 5 | 3.5 | 59.0 | DNA maintenance | 13 | 3.4 | 54.2 |
| Cytoskeleton | 4 | 2.8 | 61.8 | Cell division & differentiation | 11 | 2.9 | 57.0 |
| Ion transport | 4 | 2.8 | 64.6 | Spermatogenesis | 11 | 2.9 | 59.9 |
| Nucleotide metabolism | 4 | 2.8 | 67.4 | PTM | 10 | 2.6 | 62.5 |
| Apoptosis | 3 | 2.1 | 69.4 | Apoptosis | 9 | 2.3 | 64.8 |
| Stress response | 3 | 2.1 | 71.5 | Cytoskeleton | 9 | 2.3 | 67.2 |
| ETS | 3 | 2.1 | 73.6 | ETS | 9 | 2.3 | 69.5 |
| Ciliary activity | 3 | 2.1 | 75.7 | Adhesion | 8 | 2.1 | 71.6 |
| Redox homeostasis | 3 | 2.1 | 77.8 | Detoxification | 8 | 2.1 | 73.7 |
| Chaperone | 2 | 1.4 | 79.2 | Chaperone | 7 | 1.8 | 75.5 |
| Development | 2 | 1.4 | 80.6 | Protein transport | 7 | 1.8 | 77.3 |
| Endocytosis | 2 | 1.4 | 81.9 | Carbohydrate metabolism | 6 | 1.6 | 78.9 |
| Locomotion | 2 | 1.4 | 83.3 | Glycoprotein metabolism | 6 | 1.6 | 80.5 |
| Protein transport | 2 | 1.4 | 84.7 | Locomotion | 6 | 1.6 | 82.0 |
| Signaling | 2 | 1.4 | 86.1 | Membrane and substrate transport | 6 | 1.6 | 83.6 |
| Oxidoreductase | 2 | 1.4 | 87.5 | Endocytosis | 5 | 1.3 | 84.9 |
| Iron homeostasis | 2 | 1.4 | 88.9 | RNA metabolism | 5 | 1.3 | 86.2 |
| Neural function | 2 | 1.4 | 90.3 | Sulfur metabolism | 5 | 1.3 | 87.5 |
| Peptidoglycan synthesis | 2 | 1.4 | 91.7 | Inflammation | 4 | 1.0 | 88.5 |
| Carbohydrate metabolism | 1 | 0.7 | 92.4 | Ion transport | 4 | 1.0 | 89.6 |
| Detoxification | 1 | 0.7 | 93.1 | Stress response | 4 | 1.0 | 90.6 |
| Endocytosis | 1 | 0.7 | 93.7 | Cofactor metabolism | 3 | 0.8 | 91.4 |
| Glycoprotein metabolism | 1 | 0.7 | 94.4 | Nucleotide metabolism | 3 | 0.8 | 92.2 |
| Histone modification | 1 | 0.7 | 95.1 | Oxidoreductases | 3 | 0.8 | 93.0 |
| Inflammation | 1 | 0.7 | 95.8 | Redox homeostasis | 4 | 1.0 | 94.0 |
| Lipid metabolism | 1 | 0.7 | 96.5 | AA metabolism | 2 | 0.5 | 94.5 |
| mRNA synthesis | 1 | 0.7 | 97.2 | Histone modification | 2 | 0.5 | 95.1 |
| Oogenesis | 1 | 0.7 | 97.9 | Lipid metabolism | 2 | 0.5 | 95.6 |
| Membrane and substrate transport | 1 | 0.7 | 98.6 | Membrane function | 2 | 0.5 | 96.1 |
| Sulfur metabolism | 1 | 0.7 | 99.3 | Oogenesis | 2 | 0.5 | 96.6 |
| Tricarboxylic acid cycle | 1 | 0.7 | 100.0 | Organelle biogenesis | 2 | 0.5 | 97.1 |
|  |  |  |  | Secretion | 2 | 0.5 | 97.7 |
|  |  |  |  | Tricarboxylic acid cycle | 2 | 0.5 | 98.2 |
|  |  |  |  | Angiogenesis | 1 | 0.3 | 98.4 |
|  |  |  |  | Ca metabolism | 1 | 0.3 | 98.7 |
|  |  |  |  | Cell wall | 1 | 0.3 | 99.0 |
|  |  |  |  | Endocrine function | 1 | 0.3 | 99.2 |
|  |  |  |  | Nuclear transport | 1 | 0.3 | 99.5 |
|  |  |  |  | Sphingolipid metabolism | 1 | 0.3 | 99.7 |
|  |  |  |  | Steroid metabolism & biosynthesis | 1 | 0.3 | 100.0 |

**Supplementary Table 5.** Results of the Reactome analysis of the gill transcriptome of *L. capensis*, displaying only significantly up- or downregulated pathways (FDR < 0.1). No significantly enriched pathways were identified in the following comparisons: R24 (24 hours of reoxygenation) vs. normoxia (N), R1 (1 hour of reoxygenation) vs. hypoxia (H), and R24 vs. hypoxia. Additionally, no significantly downregulated pathways were found in the R1 vs N comparison. "# Genes found" refers to the number of identified DEGs, while "# Genes total" refers to the total number of genes in the respective pathway. Rxn – reactions. For the sake of completeness, we report all pathways identified as significantly enriched by Reactome analysis. Pathways that are specific to vertebrates and are unlikely to be relevant in mollusks are indicated in italics.

| Contrast | Direction | Pathway identifier | Pathway name | #Genes found | #Genes total | P value | FDR | #Rxn found | #Rxn total |
| --- | --- | --- | --- | --- | --- | --- | --- | --- | --- |
| H vs N | Downregulated | R-HSA-975956 | Nonsense Mediated Decay (NMD) independent of the Exon Junction Complex (EJC) | 60 | 96 | 1.11E-16 | 4.22E-15 | 1 | 1 |
| H vs N | Downregulated | R-HSA-72689 | Formation of a pool of free 40S subunits | 63 | 102 | 1.11E-16 | 4.22E-15 | 2 | 2 |
| H vs N | Downregulated | R-HSA-192823 | Viral mRNA Translation | 60 | 101 | 1.11E-16 | 4.22E-15 | 2 | 2 |
| *H vs N* | *Downregulated* | *R-HSA-156827* | *L13a-mediated translational silencing of Ceruloplasmin expression* | *64* | *112* | *1.11E-16* | *4.22E-15* | *3* | *3* |
| H vs N | Downregulated | R-HSA-72706 | GTP hydrolysis and joining of the 60S ribosomal subunit | 64 | 113 | 1.11E-16 | 4.22E-15 | 3 | 3 |
| H vs N | Downregulated | R-HSA-1799339 | SRP-dependent cotranslational protein targeting to membrane | 60 | 113 | 1.11E-16 | 4.22E-15 | 5 | 5 |
| H vs N | Downregulated | R-HSA-72649 | Translation initiation complex formation | 29 | 59 | 1.11E-16 | 4.22E-15 | 2 | 2 |
| H vs N | Downregulated | R-HSA-72702 | Ribosomal scanning and start codon recognition | 29 | 59 | 1.11E-16 | 4.22E-15 | 2 | 2 |
| H vs N | Downregulated | R-HSA-6791226 | Major pathway of rRNA processing in the nucleolus and cytosol | 71 | 183 | 1.11E-16 | 4.22E-15 | 7 | 7 |
| H vs N | Downregulated | R-HSA-927802 | Nonsense-Mediated Decay (NMD) | 61 | 117 | 1.11E-16 | 4.22E-15 | 5 | 6 |
| H vs N | Downregulated | R-HSA-72662 | Activation of the mRNA upon binding of the cap-binding complex and eIFs, and subsequent binding to 43S | 29 | 60 | 1.11E-16 | 4.22E-15 | 5 | 6 |
| H vs N | Downregulated | R-HSA-156902 | Peptide chain elongation | 60 | 90 | 1.11E-16 | 4.22E-15 | 4 | 5 |
| H vs N | Downregulated | R-HSA-975957 | Nonsense Mediated Decay (NMD) enhanced by the Exon Junction Complex (EJC) | 61 | 117 | 1.11E-16 | 4.22E-15 | 4 | 5 |
| H vs N | Downregulated | R-HSA-72613 | Eukaryotic Translation Initiation | 64 | 120 | 1.11E-16 | 4.22E-15 | 16 | 21 |
| H vs N | Downregulated | R-HSA-8868773 | rRNA processing in the nucleus and cytosol | 73 | 193 | 1.11E-16 | 4.22E-15 | 11 | 15 |
| H vs N | Downregulated | R-HSA-72737 | Cap-dependent Translation Initiation | 64 | 120 | 1.11E-16 | 4.22E-15 | 13 | 18 |
| H vs N | Downregulated | R-HSA-156842 | Eukaryotic Translation Elongation | 62 | 95 | 1.11E-16 | 4.22E-15 | 6 | 9 |
| H vs N | Downregulated | R-HSA-72312 | rRNA processing | 75 | 203 | 1.11E-16 | 4.22E-15 | 13 | 21 |
| H vs N | Downregulated | R-HSA-9711097 | Cellular response to starvation | 63 | 157 | 1.11E-16 | 4.22E-15 | 17 | 28 |
| H vs N | Downregulated | R-HSA-72764 | Eukaryotic Translation Termination | 60 | 94 | 1.11E-16 | 4.22E-15 | 3 | 5 |
| H vs N | Downregulated | R-HSA-72766 | Translation | 76 | 294 | 1.11E-16 | 4.22E-15 | 54 | 99 |
| H vs N | Downregulated | R-HSA-8953854 | Metabolism of RNA | 96 | 729 | 1.11E-16 | 4.22E-15 | 78 | 199 |
| *H vs N* | *Downregulated* | *R-HSA-168273* | *Influenza Viral RNA Transcription and Replication* | *62* | *152* | *1.11E-16* | *4.22E-15* | *5* | *13* |
| *H vs N* | *Downregulated* | *R-HSA-9735869* | *SARS-CoV-1 modulates host translation machinery* | *25* | *40* | *1.11E-16* | *4.22E-15* | *1* | *3* |
| H vs N | Downregulated | R-HSA-72695 | Formation of the ternary complex, and subsequently, the 43S complex | 28 | 52 | 1.11E-16 | 4.22E-15 | 1 | 3 |
| H vs N | Downregulated | R-HSA-9633012 | Response of EIF2AK4 (GCN2) to amino acid deficiency | 61 | 102 | 1.11E-16 | 4.22E-15 | 5 | 16 |
| H vs N | Downregulated | R-HSA-2408557 | Selenocysteine synthesis | 60 | 94 | 1.11E-16 | 4.22E-15 | 2 | 7 |
| H vs N | Downregulated | R-HSA-9010553 | Regulation of expression of SLITs and ROBOs | 64 | 172 | 1.11E-16 | 4.22E-15 | 5 | 19 |
| H vs N | Downregulated | R-HSA-2408522 | Selenoamino acid metabolism | 64 | 118 | 1.11E-16 | 4.22E-15 | 5 | 23 |
| *H vs N* | *Downregulated* | *R-HSA-168255* | *Influenza Infection* | *63* | *172* | *1.11E-16* | *4.22E-15* | *11* | *58* |
| H vs N | Downregulated | R-HSA-376176 | Signaling by ROBO receptors | 65 | 218 | 1.11E-16 | 4.22E-15 | 7 | 59 |
| H vs N | Downregulated | R-HSA-71291 | Metabolism of amino acids and derivatives | 78 | 376 | 1.11E-16 | 4.22E-15 | 22 | 248 |
| *H vs N* | *Downregulated* | *R-HSA-9754678* | *SARS-CoV-2 modulates host translation machinery* | *25* | *53* | *3.33E-16* | *1.23E-14* | *1* | *6* |
| H vs N | Downregulated | R-HSA-422475 | Axon guidance | 71 | 558 | 6.15E-12 | 2.21E-10 | 26 | 297 |
| H vs N | Downregulated | R-HSA-2262752 | Cellular responses to stress | 86 | 779 | 4.41E-11 | 1.54E-09 | 93 | 486 |
| H vs N | Downregulated | R-HSA-9675108 | Nervous system development | 71 | 584 | 4.60E-11 | 1.56E-09 | 26 | 323 |
| *H vs N* | *Downregulated* | *R-HSA-9692914* | *SARS-CoV-1-host interactions* | *27* | *109* | *5.55E-11* | *1.83E-09* | *6* | *48* |
| H vs N | Downregulated | R-HSA-8953897 | Cellular responses to stimuli | 86 | 793 | 1.06E-10 | 3.40E-09 | 93 | 517 |
| *H vs N* | *Downregulated* | *R-HSA-9678108* | *SARS-CoV-1 Infection* | *30* | *154* | *1.33E-09* | *4.11E-08* | *12* | *156* |
| *H vs N* | *Downregulated* | *R-HSA-9705683* | *SARS-CoV-2-host interactions* | *32* | *221* | *3.29E-07* | *9.87E-06* | *10* | *67* |
| H vs N | Downregulated | R-HSA-1430728 | Metabolism | 160 | 2150 | 7.06E-07 | 2.12E-05 | 237 | 2044 |
| *H vs N* | *Downregulated* | *R-HSA-9694516* | *SARS-CoV-2 Infection* | *36* | *316* | *1.35E-05* | *3.92E-04* | *17* | *208* |
| H vs N | Downregulated | R-HSA-9824446 | Viral Infection Pathways | 79 | 980 | 6.95E-05 | 0.00194584 | 89 | 644 |
| H vs N | Downregulated | R-HSA-6790901 | rRNA modification in the nucleus and cytosol | 11 | 60 | 3.74E-04 | 0.01046335 | 4 | 8 |
| H vs N | Downregulated | R-HSA-163200 | Respiratory electron transport, ATP synthesis by chemiosmotic coupling, and heat production by uncoupling proteins. | 17 | 127 | 4.50E-04 | 0.0121625 | 17 | 28 |
| H vs N | Downregulated | R-HSA-1253288 | Downregulation of ERBB4 signaling | 4 | 11 | 0.00277726 | 0.07220863 | 5 | 5 |
| H vs N | Downregulated | R-HSA-611105 | Respiratory electron transport | 13 | 103 | 0.00332034 | 0.08358227 | 14 | 19 |
| H vs N | Downregulated | R-HSA-6799198 | Complex I biogenesis | 9 | 57 | 0.00334329 | 0.08358227 | 11 | 13 |
| H vs N | Upregulated | R-HSA-1428517 | The citric acid (TCA) cycle and respiratory electron transport | 13 | 238 | 6.33E-05 | 0.06294941 | 24 | 67 |
| R1 vs N | Upregulated | R-HSA-5675482 | Regulation of necroptotic cell death | 6 | 39 | 4.72E-05 | 0.0465962 | 9 | 21 |
| R1 vs N | Upregulated | R-HSA-5213460 | RIPK1-mediated regulated necrosis | 6 | 45 | 1.03E-04 | 0.0507073 | 10 | 29 |
| R1 vs N | Upregulated | R-HSA-168927 | TICAM1, RIP1-mediated IKK complex recruitment | 4 | 19 | 2.79E-04 | 0.09183038 | 1 | 3 |

**Supplementary Table 6.** Results of the Reactome analysis of the digestive gland transcriptome of *L. capensis*, displaying only significantly up- or downregulated pathways (FDR < 0.1). No significantly downregulated pathways were identified in the following comparisons: hypoxia (H) vs. normoxia (N), R24 (24 hours of reoxygenation) vs. N. No significantly downregulated pathways were identified in the following comparisons: R24 v. N and R24 vs. A. "# Genes found" refers to the number of identified DEGs, while "# Genes total" refers to the total number of genes in the respective pathway. Rxn – reactions.

| Contrast | Direction | Pathway identifier | Pathway name | #Genes found | #Genes total | P value | FDR | #Rxn found | #Rxn total |
| --- | --- | --- | --- | --- | --- | --- | --- | --- | --- |
| H vs N | Upregulated | R-HSA-8851708 | Signaling by FGFR2 IIIa TM | 5 | 23 | 2.62E-04 | 0.0965289 | 2 | 2 |
| H vs N | Upregulated | R-HSA-1679131 | Trafficking and processing of endosomal TLR | 4 | 13 | 3.01E-04 | 0.0965289 | 2 | 7 |
| H vs N | Upregulated | R-HSA-1839126 | FGFR2 mutant receptor activation | 6 | 38 | 3.52E-04 | 0.0965289 | 17 | 18 |

**Supplementary Table 7.** Significantly enriched pathways identified through MetaboAnalyst analysis of *L. capensis* gill metabolome profiles under normoxic conditions versus 1 hour of reoxygenation. Only pathways with a False Discovery Rate (FDR) < 0.1 are included. "Total Compounds" refers to the total number of compounds associated with each pathway, while "Hits" indicates the number of metabolites detected in *L. capensis* gills that are linked to each pathway.

| Pathway | Total compounds | Hits | Raw p | FDR | Impact |
| --- | --- | --- | --- | --- | --- |
| Citrate cycle (TCA cycle) | 20 | 4 | 0.00062 | 0.0096 | 0.18 |
| Butanoate metabolism | 14 | 4 | 0.00876 | 0.0761 | 0.2 |
| Pyruvate metabolism | 23 | 3 | 0.01187 | 0.0761 | 0.18 |
| Cysteine and methionine metabolism | 32 | 5 | 0.01227 | 0.0761 | 0.21 |


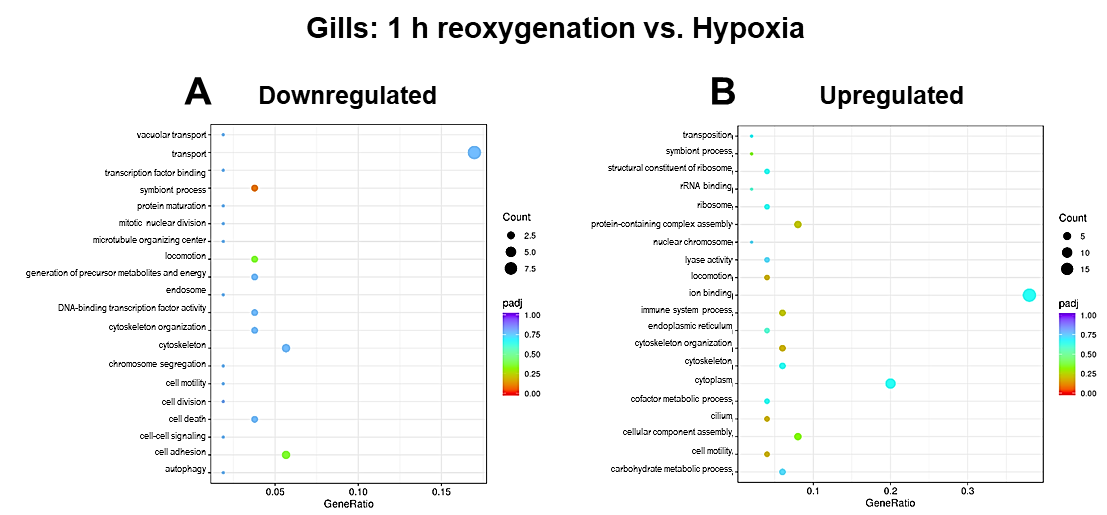
**Supplementary Figure 1.** GO pathway enrichment in the gills of *L. capensis* after 1 h of reoxygenation relative to the hypoxic state. Color indicates the significance (p_adj_) and size of the symbols – the number of DEGs found in the respective pathway. X axis shows Gene Ratio for each pathway, depicting the ratio of differentially expressed genes to all genes for this GO term.


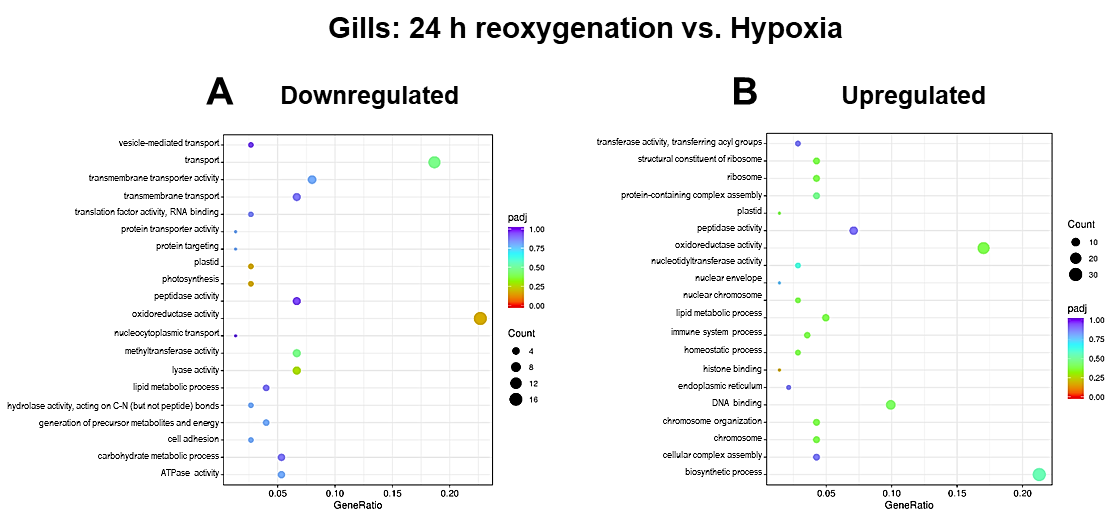
**Supplementary Figure 2.** GO pathway enrichment in the gills of *L. capensis* after 24 h of reoxygenation relative to the hypoxic state. Color indicates the significance (p_adj_) and size of the symbols – the number of DEGs found in the respective pathway. X axis shows Gene Ratio for each pathway, depicting the ratio of differentially expressed genes to all genes for this GO term.


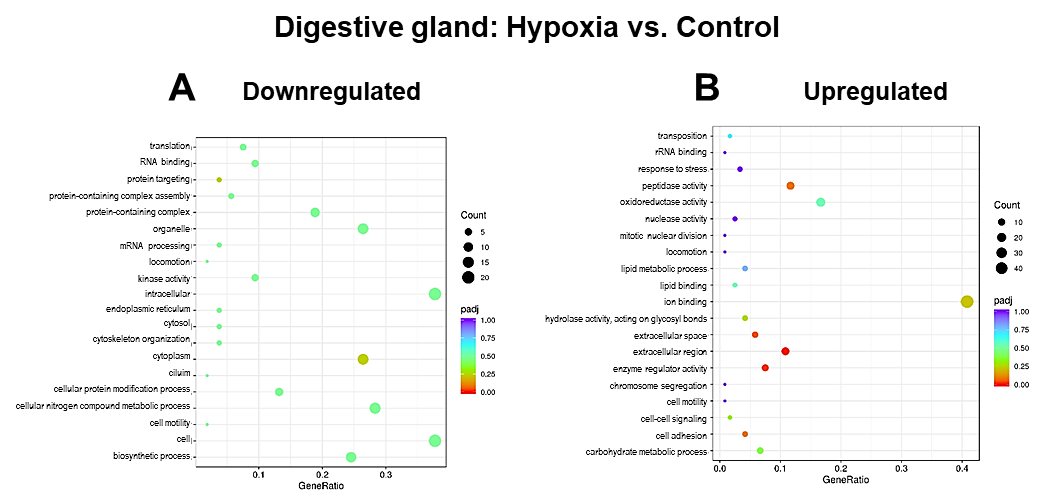
**Supplementary Figure 3.** GO pathway enrichment in the hypoxic digestive gland of *L. capensis* relative to the normoxic control. Color indicates the significance (p_adj_) and size of the symbols – the number of DEGs found in the respective pathway. X axis shows Gene Ratio for each pathway, depicting the ratio of differentially expressed genes to all genes for this GO term.

**Supplementary Figure 4.** GO pathway enrichment in the digestive gland of *L. capensis* after 24 h of reoxygenation relative to the normoxic control. Color indicates the significance (p_adj_) and size of the symbols – the number of DEGs found in the respective pathway. X axis shows Gene Ratio for each pathway, depicting the ratio of differentially expressed genes to all genes for this GO term.


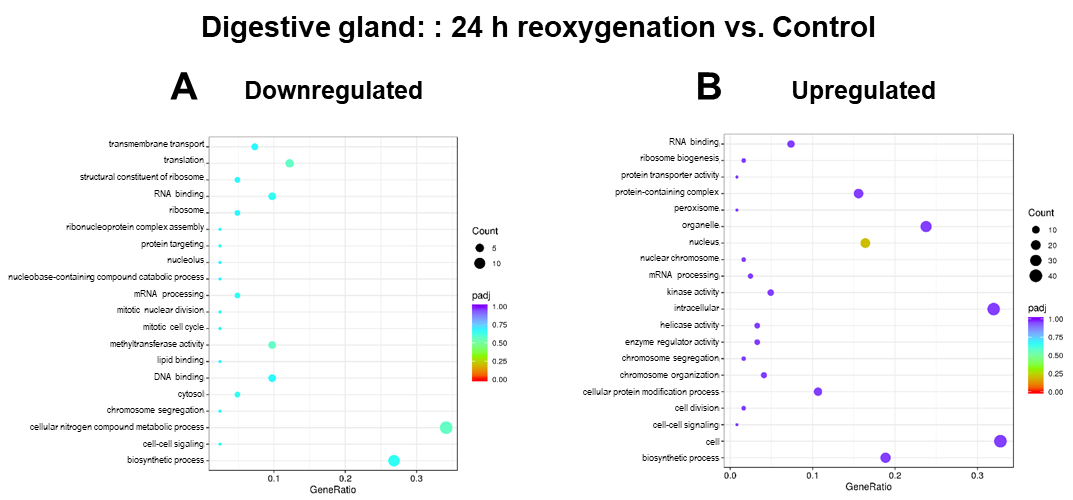


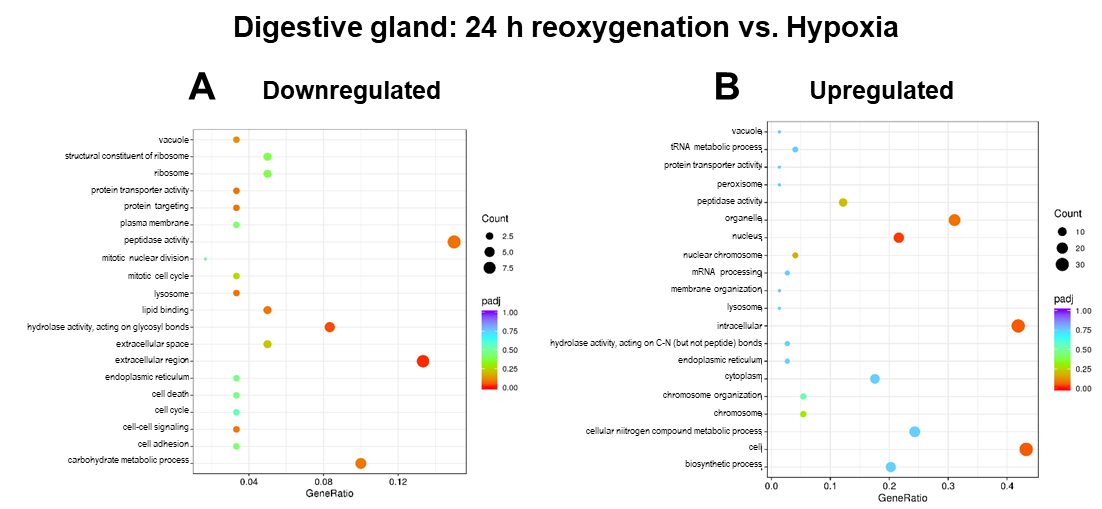
**Supplementary Figure 5.** GO pathway enrichment in the digestive gland of *L. capensis* after 24 h of reoxygenation relative to the hypoxic state. Color indicates the significance (p_adj_) and size of the symbols – the number of DEGs found in the respective pathway. X axis shows Gene Ratio for each pathway, depicting the ratio of differentially expressed genes to all genes for this GO term.
